# Protein synthesis inhibitor omacetaxine is effective against hepatocellular carcinoma

**DOI:** 10.1172/jci.insight.138197

**Published:** 2021-06-22

**Authors:** Ling Li, Gilad Halpert, Michael G. Lerner, Haijie Hu, Peter Dimitrion, Matthew J. Weiss, Jin He, Benjamin Philosophe, Richard Burkhart, William R. Burns, Russell N. Wesson, Andrew MacGregor Cameron, Christopher L. Wolfgang, Christos Georgiades, Satomi Kawamoto, Nilofer S. Azad, Mark Yarchoan, Stephen J. Meltzer, Kiyoko Oshima, Laura M. Ensign, Joel S. Bader, Florin M. Selaru

**Affiliations:** 1 Division of Gastroenterology and Hepatology and; 2 Center for Nanomedicine at the Wilmer Eye Institute, Department of Ophthalmology, Johns Hopkins University School of Medicine, Baltimore, Maryland, USA.; 3 Department of Physics and Astronomy, Earlham College, Richmond, Indiana, USA.; 4 Department of Surgery, Johns Hopkins University School of Medicine, Baltimore, Maryland, USA.; 5 Department of Surgery, NYU Grossman School of Medicine, New York, New York, USA.; 6 Department of Radiology and; 7 Department of Oncology, Sidney Kimmel Cancer Center, Johns Hopkins University School of Medicine, Baltimore, Maryland, USA.; 8 Department of Pathology and; 9 Department of Biomedical Engineering, Johns Hopkins University, Baltimore, Maryland, USA.

**Keywords:** Hepatology, Oncology, Drug therapy, Liver cancer

## Abstract

Hepatocellular carcinoma (HCC) is the sixth most common and the fourth most deadly cancer worldwide. The development cost of new therapeutics is a major limitation in patient outcomes. Importantly, there is a paucity of preclinical HCC models in which to test new small molecules. Herein, we implemented potentially novel patient-derived organoid (PDO) and patient-derived xenografts (PDX) strategies for high-throughput drug screening. Omacetaxine, an FDA-approved drug for chronic myelogenous leukemia (CML), was found to be a top effective small molecule in HCC PDOs. Next, omacetaxine was tested against a larger cohort of 40 human HCC PDOs. Serial dilution experiments demonstrated that omacetaxine is effective at low (nanomolar) concentrations. Mechanistic studies established that omacetaxine inhibits global protein synthesis, with a disproportionate effect on short–half-life proteins. High-throughput expression screening identified molecular targets for omacetaxine, including key oncogenes, such as PLK1. In conclusion, by using an innovative strategy, we report — for the first time to our knowledge — the effectiveness of omacetaxine in HCC. In addition, we elucidate key mechanisms of omacetaxine action. Finally, we provide a proof-of-principle basis for future studies applying drug screening PDOs sequenced with candidate validation in PDX models. Clinical trials could be considered to evaluate omacetaxine in patients with HCC.

## Introduction

Hepatocellular carcinoma (HCC) is among the most frequent malignancies worldwide, with approximately 765,000 new cases each year (1, 2). The WHO estimates that, by 2030, more than 1 million people will die annually due to HCC (3). In the United States, the death rate due to HCC has seen a sharp 43% increase from 2000 to 2016, explained by an increase in incidence. Moreover, survival in the United States is poor, with only 18% surviving 5 years (3). The only curative approach to HCC is surgical resection or liver transplantation (4). Unfortunately, more than 70% of cases are diagnosed at advanced stages, when surgery is no longer an option (5). Moreover, approximately 70% of HCC patients suffer recurrence and/or metastasis within 5 years after surgical intervention (6). For advanced HCC, systemic agents sorafenib and lenvantinib, as well as the second-line agents regorafenib, cabozantinib, and ramucirumab, achieve only fairly low response rates (7–10). Programmed death 1 (PD-1) inhibitors have recently shown clinical activity as second-line treatment but failed to improve survival as single-agent in both first- or second-line settings (11). However, a combination of an anti-VEGF antibody with an anti–PD-1 or anti–PD-L1 agent seemed attractive due to the hypothesized reduction in VEGF-induced immunosuppression. Indeed, a phase III study demonstrated that a combination of atezolizumab (PD-L1 inhibitor) and bevacizumab (monoclonal antibody anti-VEGF) improved the median progression-free survival to 6.8 months from 4.3 months with sorafenib (11).

The development of effective systemic therapies against HCC has been hindered, at least in part, by a paucity of representative HCC models. Recent work by us and others demonstrated that 3-dimensional (3D) human patient-derived organoid (PDO) models accurately capture original HCC tissue architecture while maintaining the genomic, epigenetic, and molecular heterogeneity features of original tumor source material, even after long-term expansion (12–17). In addition, HCC PDOs are powerful tools for drug screening, as we showed in previous studies (12, 15).

Omacetaxine mepusuccinate (formerly known as homoharringtonine) is approved by the FDA for relapsed/refractory or accelerated-phase chronic myelogenous leukemia (CML) that is resistant to 2 or more tyrosine kinase inhibitors (TKIs) (18). Unlike TKIs, however, omacetaxine does not bind to BCR-ABL, nor is it affected by resistance-inducing BCR-ABL mutations (19–22). Omacetaxine acts by blocking global protein synthesis, with a disproportionate effect on proteins, such as BCR-ABL, that have short half-lives and depend on continuous protein synthesis (18). The toxicity profile for omacetaxine is favorable at its FDA-approved dosage. Principal side effects include hematologic depletion (thrombocytopenia, anemia, and neutropenia) and diarrhea, while infectious complications occur in approximately 5% of patients (23). Omacetaxine is therefore valuable for CML and is currently being investigated as a single agent, as well as in multidrug combinations in CML patients (18, 24). Currently, omacetaxine comprises the only agent in its class that possesses demonstrated clinical activity (18). Furthermore, there have been no published preclinical or clinical studies investigating the efficacy of omacetaxine in any solid cancers. In the current manuscript, we newly report that omacetaxine is effective in a large cohort of HCC PDOs, as well as in vivo in patient-derived xenograft (PDX) models. In addition, we characterize its mechanism of action in vitro and in vivo.

## Results

### Establishment and validation of human HCC PDOs.

We previously reported the establishment of 10 HCC PDO lines (15). Average cell survival across all 10 PDO lines following treatment with omacetaxine at a 10 μM concentration was 5.2%. In the current study, to validate these preliminary findings, we explored the effectiveness of omacetaxine in a much larger HCC PDO library. To this end, we established 30 potentially new HCC PDO lines from primary human tumors, amassing a total of 40 human HCC PDO lines. In some patients, tumor mass was sufficiently large to permit the establishment of more than 1 HCC PDO line per tumor. In large HCC tumors, geographically distinct areas displayed genetic as well as drug-response heterogeneity, partially reported previously (15). For this reason, we included all 40 HCC PDO lines. Clinical and histopathologic information for these PDOs is summarized in Table 1.

### Establishment and validation of human HCC PDXs.

**Figure 1:**
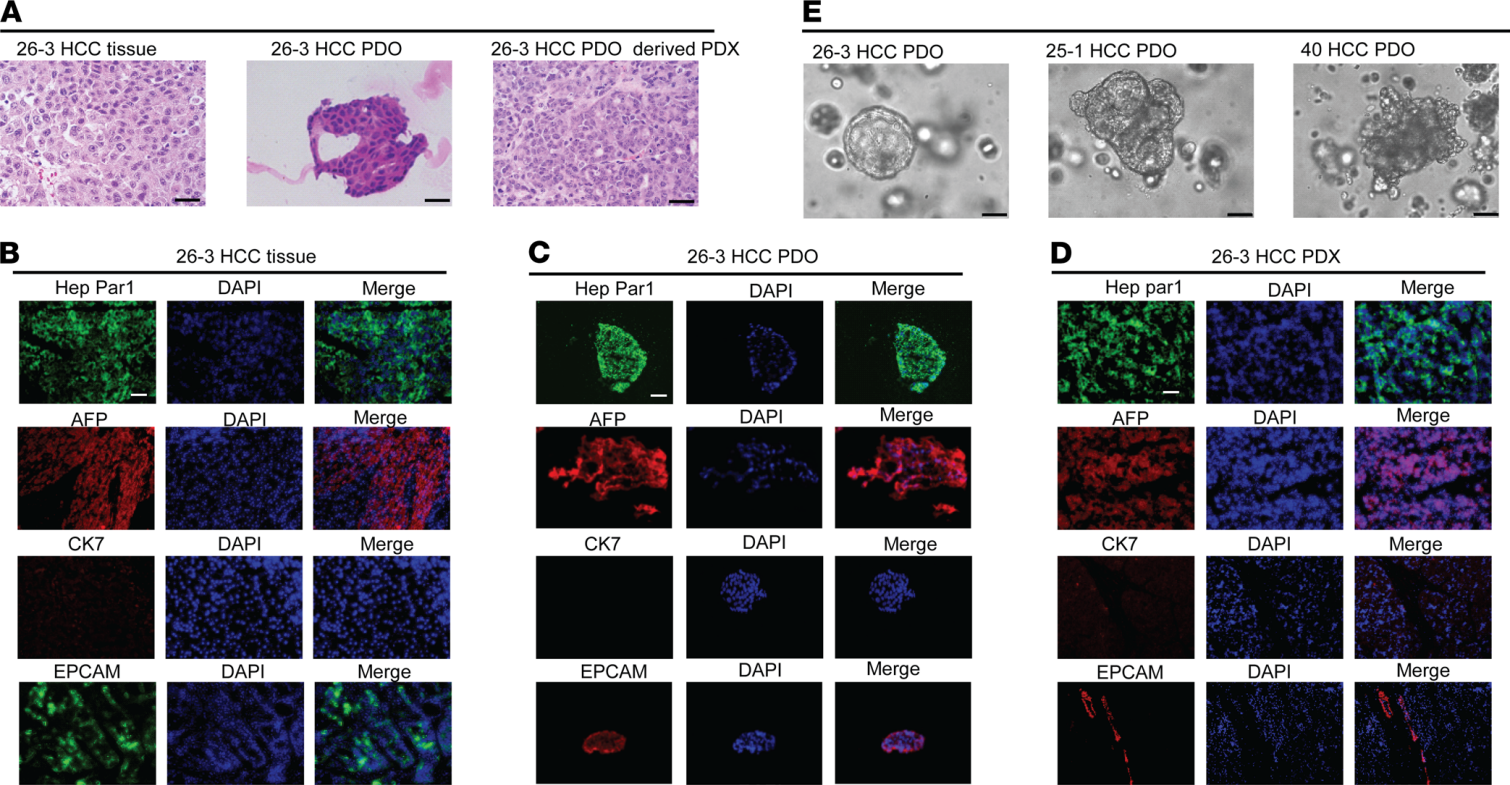
Validation of HCC PDO and PDX models. (**A**) H&E staining demonstrates a similar morphology/trabecular pattern in the human tissue 26-3 HCC, matched 26-3 HCC PDO, and HCC PDX26. (**B**–**D**) Clinical diagnostic immunofluorescence staining on the primary human HCC tissue, as well as on the matched PDO and PDX, demonstrates similar positivity for Hep Par1, AFP, CK7, and EPCAM. (**E**) Bright-field images of 3 distinct HCC PDO lines. Scale bars: 25 µM (**A** and **E**), 50 µM (**B**–**D**).

We developed 2 distinct HCC PDX models to establish in vivo drug effectiveness documented in human HCC PDOs in vitro. H&E staining results in 1 primary HCC tumor specimen, its matched PDO, and its matched PDX (Figure 1A). H&E staining of primary human HCC tissue (Figure 1A) revealed neoplastic cells arranged in a trabecular pattern, without glandular architecture. H&E staining of the matched PDO, as well as the matched PDX, showed clusters of neoplastic cells forming trabeculae, morphologically similar to the primary HCC (Figure 1A). Next, standard clinical diagnostics were performed, including immunofluorescence staining for the hepatocellular marker HepPar1, the hepatocellular marker AFP, the hepatic progenitor/stem cell marker EPCAM, and the cholangiocyte marker CK7. As shown in Figure 1, B–D, HCC cells from the primary tumor, matched PDO, and matched PDX all displayed similar staining: positive for hepatocellular and HCC markers, focally positive for hepatocellular progenitor/stem cell markers, and negative for cholangiocyte markers. These findings confirmed the diagnosis of HCC while excluding cholangiocarcinoma or other types of malignancy. Figure 1E displays phase microscopy images of 3 different HCC PDOs demonstrating the expected heterogeneity in appearance (ranging from cystic to compact/spheroid type) observed in other studies (12, 25).

### Exome alterations in HCC PDO and PDX cells.

To better characterize the PDO and PDX lines we established, we sought to perform exome sequencing. We found strong evidence for mutations in known and common drivers in HCC. For example, 2 driver mutations in p53 have high scores: p.C199Y has the highest CHASMplus score of all variants, 0.865, and is seen in HCC PDO40 (CHASMplus scores range from 0 to 1, with higher scores meaning higher likelihood of a mutation to be cancer driver mutation). CHASMplus is a computational method to predict driver missense mutations, which is uniquely powered to identify rare driver mutations within the long-tail (Supplemental Table 1; supplemental material available online with this article; https://doi.org/10.1172/jci.insight.138197DS1). p.P33R has a score of 0.247 and is seen in HCC PDO26, -30, -34, -36, -40, -65, -67, and -77. In addition, p.P33R was also seen in PDX26, which is to be expected since HCC PDX26 was established by implantation of HCC PDO26 in NOD–SCID–IL-2Rγ chain–deficient (NSG) mice. This finding argues that PDX models established from PDO cells are representative for the cells of origin. All samples except for PDX50 had at least 1 APOB mutation scoring greater than zero, and PDX26, as well as PDO30, –34-4, and –36-3 had 1 ranked 0.1 or higher. Similarly, all samples except for PDX50 had at least 1 RP1L1 mutation scoring above zero. At least 1 IL6ST mutation ranked 0.1 or higher in several samples: PDX26, and PDO40, -59, –65-2, –67-1, –67-2, and –80-4. Two samples had more than 1 MET mutation ranked 0.1 or higher; 3 more samples had 1 MET mutation scoring above zero. All samples except for PDX50 and PDO67-1, –67-2, and –71-1 exhibit at least 1 CCDN1 mutation, though none of these mutations are scored as functional by CHASMplus. There was less evidence for other common drivers. For example, 26% of HCCs have CTTNB1 mutations (26), while we see only 1 synonymous exonic mutation (p.A457A) in PDO83.

PDX50 exhibited a unique mutational signature. It displayed 31 exonic ARID1A mutations, of which 3 scored higher than 0.1 (p.T118P, p.P153A, and p.S1197P). Based on this score, we hypothesize that ARID1A could be a driver gene in this patient. Exonic mutations in APOB, RP1L1, and p53 are notably absent, as are several other exonic mutations found in the other samples. These data underline the great diversity of genetic alterations seen in HCC samples who appear otherwise fairly similar in regards to standard clinical pathology staining.

PDX26, which was established by implanting PDO26-3 cells into NSG mice, appears more similar to the majority of PDOs. PDX26 displayed mutations in IL6ST, as well as APOB, ARID1A, AXIN1, EEF1A1, RP1L1, p53, and TSC1.

### Identification and validation of omacetaxine as an effective anti-HCC agent.

**Figure 2:**
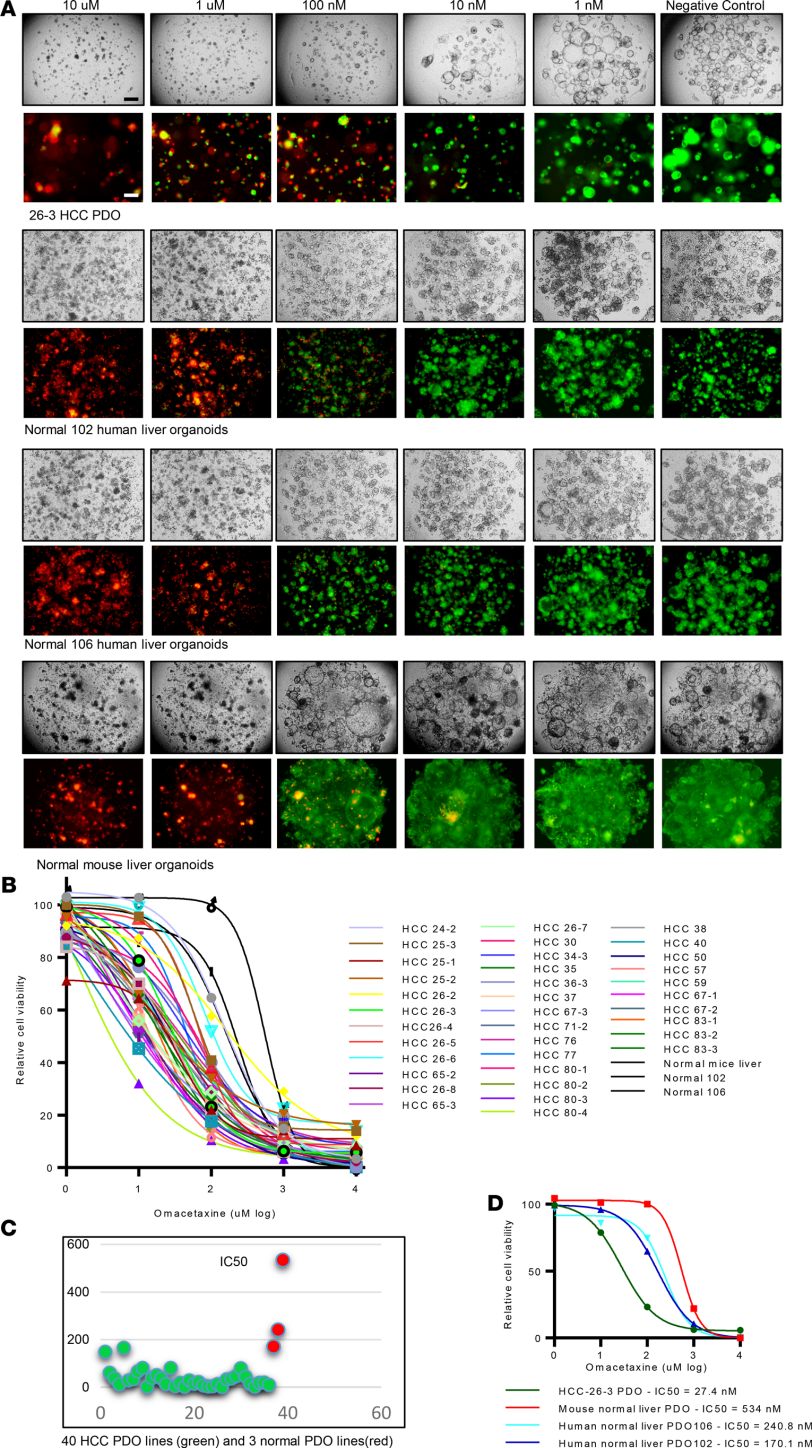
Omacetaxine is effective in human HCC PDOs. (**A**) Live/dead cell staining (live, green; dead, red) of PDO lines treated with omacetaxine at dose-limiting concentrations. Note that, at 100 nM, normal liver cells stained mostly green (alive), while HCC cells stained mostly red (dead). Scale bars: 200 µM. (**B**) IC_50_ curves of omacetaxine across all 40 PDO lines and 2 human normal liver PDO lines, as well as 1 mouse normal liver PDO line. The *x* axis shows omacetaxine dose (from 1 nM to 10 μM), and the *y* axis shows Cell viability. The 3 curves to the right of the graphs (most resistant to omacetaxine) are the normal liver PDO lines, in line with their relative resistance to omacetaxine. (**C**) IC_50_ values for omacetaxine are shown for all 40 HCC PDOs (green dots in the figure) as well 3 normal liver PDO lines (red dots in the figure). The *x* axis shows each of the PDO lines, and the *y* axis shows the IC_50_ value for omacetaxine for each HCC PDO and normal liver PDOs. (**D**) IC_50_ curves for 2 human normal liver PDO lines, as well as 1 mouse normal liver PDO line. Data were obtained from 3 replicated experiments.

Supplemental Figure 1 displays a representative bright-field microscopy image of one of the HCC PDO lines treated with 129 drugs. In the figure, each panel represents the image obtained at 96 hours of a PDO well treated with each of the 129 drugs at a concentration of 10 μM. Based on these images, it appears that certain drugs, such as omacetaxine (highlighted in yellow in Supplemental Figure 1), are cytotoxic, while others allow unrestricted organoid growth. The top effective drugs were proteasome inhibitors (such as bortezomib) (15) or HDAC inhibitors (15). Omacetaxine, one of the top effective drugs, is the only FDA-approved cancer drug that is a global protein synthesis inhibitor. Due to the potential of identifying of a new class of drugs in HCC, we chose to further pursue omacetaxine in the current study. Next, we tested omacetaxine at decreasing concentrations (10 μM, 1 μM, 100 nM, 10 nM, and 1 nM) in all 40 HCC PDO lines, as well as in 2 human normal liver PDO lines and 1 mouse normal liver organoid line to calculate its half-maximal inhibitory concentration (IC_50_). As shown in Figure 2A, omacetaxine displayed a definitive dose response (i.e., with increasing concentrations resulting in fewer live cells and more dead cells). Figure 2B displays IC_50_ curves for each of the 40 HCC PDOs, as well as for the 2 human and 1 mouse normal liver organoid lines. While there was variability, which was expected due to functional (drug response) heterogeneity among patients, the IC_50_ curves demonstrated that all 40 HCC PDOs were sensitive to omacetaxine at nanomolar concentrations. The calculated average IC_50_ across all 40 HCC PDO lines was 31.4 nM, which is less than the maximal concentration (C_max_) of omacetaxine in FDA-qualifying human studies of 46 nM (25.1 ng/mL). The IC_50_ values for all the 40 HCC PDO lines are shown in Figure 2C. Importantly, normal liver organoid lines had an IC_50_ of 170, 240, and 534 nm (Figure 2D). These concentrations are significantly higher than C_max_ in clinical trials of omacetaxine (46 nm) in CML patients, which explains why there were no observed liver toxicities in these CML patients. It is furthermore reassuring that the average IC_50_ in HCC organoid lines (31.4 nm) is 5.5- to 17-fold smaller than the IC_50_ of omacetaxine in normal liver organoid lines. These data indicate that omacetaxine has a wide therapeutic index that makes it an attractive option for patients with HCC.

### Omacetaxine represses growth and increases apoptosis in HCC PDOs.

**Figure 3:**
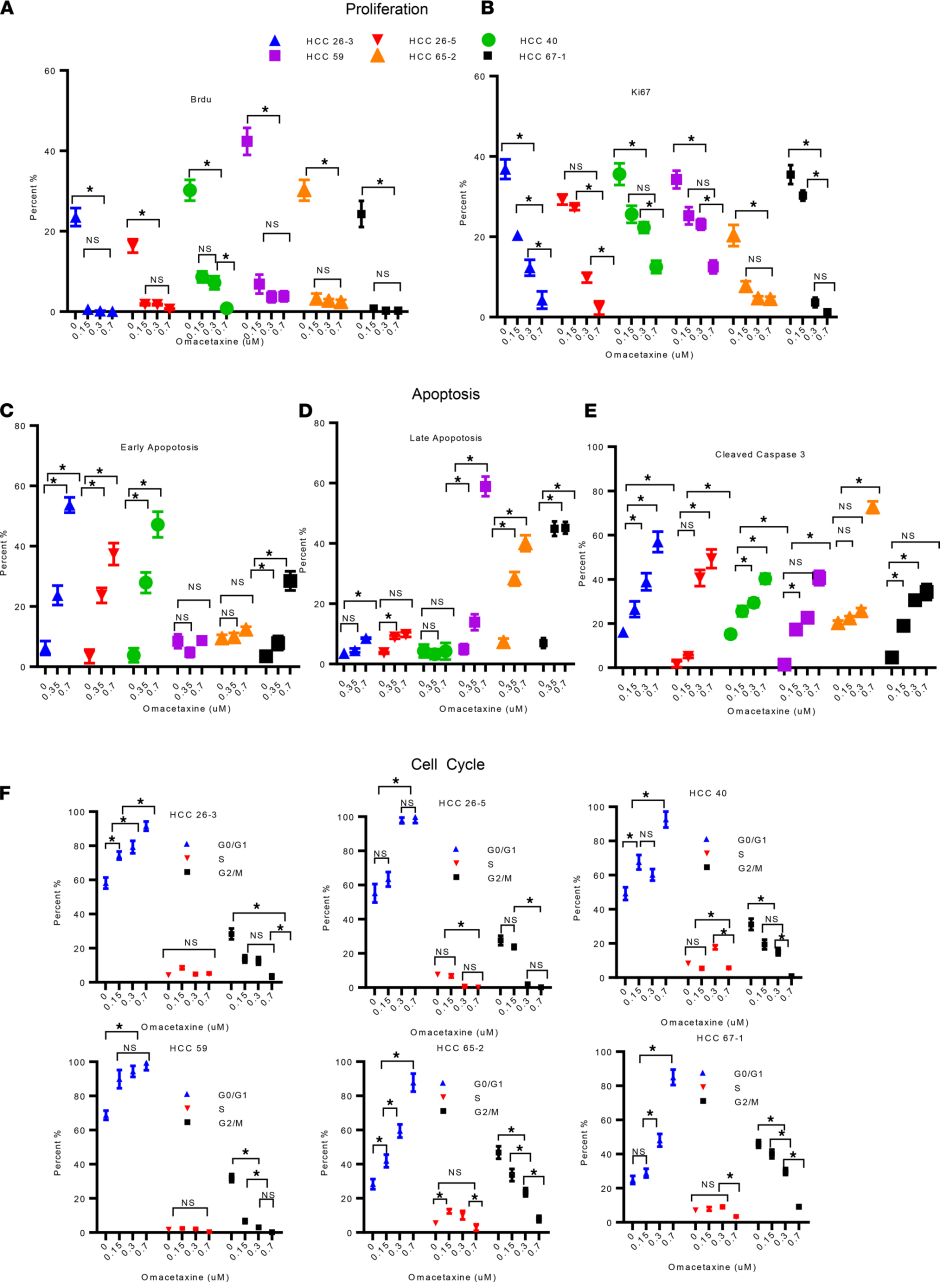
Omacetaxine impacts proliferation, apoptosis, and cell cycle. Six HCC PDO lines were treated with omacetaxine at various concentrations (negative control: 0 μM, 0.15 μM, 0.30 μM, and 0.7 μM). (**A**) BrdU incorporation demonstrates statistically significantly lower proliferation with omacetaxine in each of the 6 human HCC PDO lines tested. Primary FACS data are shown in Supplemental Figure 2. (**B**) Ki-67 staining verifies decreasing proliferation with omacetaxine treatment in each of the 6 human HCC PDO lines. Primary FACS data are shown in Supplemental Figure 3. (**C** and **D**) Annexin V/7AAD staining (**C**). Bar graphs display the effects of omacetaxine on early apoptosis (**C**) and late apoptosis (**D**). Primary FACS data are shown in Supplemental Figure 4. (**E**) Flow cytometry analysis of cleaved caspase 3 expression. Primary FACS data are shown in Supplemental Figure 5. (**F**) Effects of omacetaxine on cell cycle. Histograms of PI staining are shown in Supplemental Figure 6. Data are shown as the mean ± SEM from 3 independent experiments. Two-tailed Student’s *t* test was used for all the statistical analysis. *P < 0.01.

FACS demonstrated that omacetaxine inhibited incorporation of BrdU into newly synthesized DNA during S-phase. In fact, omacetaxine inhibited proliferation in all 6 HCC PDO lines tested (Figure 3A and Supplemental Figure 2). To further elucidate omacetaxine’s effects on cancer cell proliferation and cell cycle progression, we performed FACS analysis of Ki-67 protein. Ki-67 is a nuclear protein expressed in proliferating cells in all phases of the cell cycle except G0 (27, 28). Furthermore, elevated Ki-67 expression has been linked to poorer disease-free and overall survival in HCC patients (29). As shown in Figure 3B and Supplemental Figure 3, omacetaxine uniformly reduced Ki-67 expression in all 6 HCC PDO lines tested. Moreover, flow cytometric analysis of Annexin V and 7-Aminoactinomycin D (7AAD) demonstrated that early apoptosis, late apoptosis, or both were augmented by omacetaxine in all 6 HCC PDO lines (Figure 3, C and D, and Supplemental Figure 4). Because caspase 3 is a known participant in apoptosis, we measured activated cleaved caspase 3. Indeed, cleaved caspase 3 was induced by omacetaxine in all 6 HCC PDO lines tested (Figure 3E and Supplemental Figure 5). Next, we performed cell cycle analysis to determine at which phase cells are arrested by omacetaxine. As shown in Figure 3F and Supplemental Figure 6, omacetaxine induced G0/G1 arrest, likely explaining its effects on cell proliferation noted above. These effects were dose dependent, which further reaffirms the validity and reproducibility of our findings.

### Omacetaxine inhibits protein synthesis.

**Figure 4:**
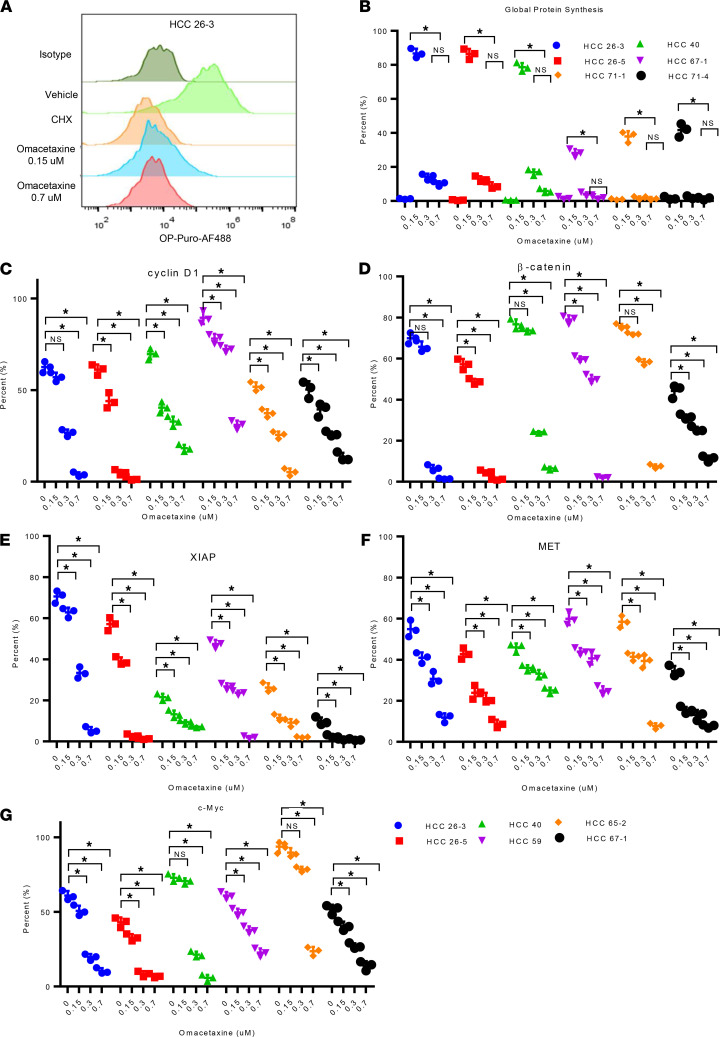
Omacetaxine acts through inhibition of oncoprotein synthesis. (**A**) The histogram demonstrates the effects of omacetaxine on global protein synthesis. Vehicle is the negative control. CHX is the positive control. Increasing concentrations of omacetaxine induce increasing protein synthesis inhibition. (**B**) Flow cytometry plots demonstrate that omacetaxine inhibits protein synthesis in each of the 6 HCC PDO lines tested. The FACS plot data are shown in Supplemental Figure 7. (**C**–**G**) Omacetaxine inhibits the expression of the following 5 oncoproteins: c-MYC, β-catenin, XIAP, MET, and cyclin D1; the flow cytometry gating plot data were displayed in Supplemental Figures 8–12. Data are shown as mean ± SEM from 3 independent experiments. 1-way ANOVA analysis was used for the statistical analysis. * *P* < 0.01.

Previous studies, mostly in acute myelogenous leukemia (AML)/CML disease models, showed that omacetaxine inhibits protein synthesis by blocking formation of the first peptide bond during polypeptide synthesis (18, 30). In AML, short-lived proteins with high synthesis rates are preferentially affected by global protein synthesis inhibition from omacetaxine (30). Furthermore, certain AML subtypes (such as FLT3-ITD AML) appear to become addicted to a high protein synthesis rate, which explains (at least in part) their sensitivity to omacetaxine (30). We hypothesized that the effectiveness of omacetaxine in HCC PDOs, resulting in reduced proliferation and increased apoptosis as noted above, is explained at least in part by global protein synthesis inhibition. To test this hypothesis, relative protein synthesis rates in 6 HCC PDOs were measured by O-propargyl-puromycin (OP-Puro) incorporation assay (EZClick, Global Protein Synthesis Assay; Figure 4A). Supplemental Figure 8 shows FACS plots for each of the 6 HCC PDOs when treated with isotype control, negative control, or omacetaxine at 0.15 μM and 0.7 μM. Figure 4B and Supplemental Figure 7 demonstrate that omacetaxine dramatically and significantly inhibited global protein synthesis at each of the 2 concentrations tested. Of interest, the higher concentration of 0.7 μM did not induce any further inhibition of protein synthesis versus the 0.15 μM concentration.

### Omacetaxine inhibits protein synthesis in HCC.

cMYC, β-catenin, cyclin D1, XIAP, and MET are short–half-life proteins that have previously been reported to be downregulated by omacetaxine in CML (18, 31, 32). To investigate if omacetaxine also affects these proteins in HCC — explaining, at least in part, its effectiveness — 6 HCC PDO lines were treated with omacetaxine, and levels of these 5 proteins were assayed via FACS at 96 h (Figure 4, C–G, and Supplemental Figures 8–12). These experiments showed that all 5 proteins were inhibited by omacetaxine. In conclusion, not only does omacetaxine inhibit global protein synthesis in HCC, but levels of specific short–half-life proteins are also decreased.

### Omacetaxine inhibits oncogene transcription in HCC.

**Figure 5:**
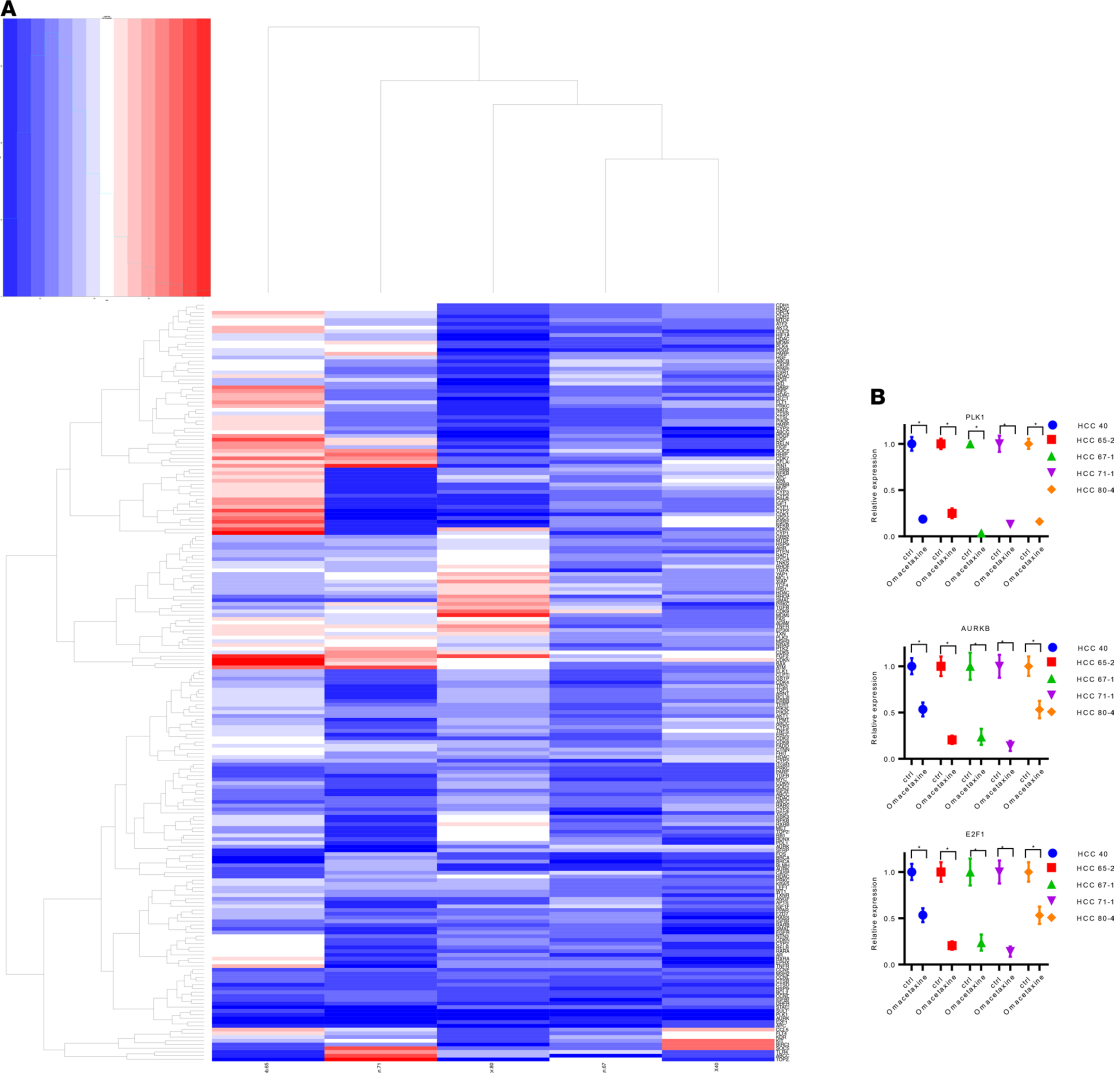
Heatmap of gene expression ratio in omacetaxine-treated PDOs compared with control. (**A**) The *x* axis shows 5 PDO lines, and the *y* axis shows a 203-gene expression ratio of omacetaxine-treated PDO/untreated PDO. Red represents upregulating gene expression, and blue represents downregulating gene expression (treated versus untreated PDO). This groups of genes includes PLK1, AURKB, and E2F1. (**B**) The relative gene expression of PLK1, AURKB, and E2F1 across the 5 human HCC PDOs. Omac, omacetaxine; ctrl, control. The figure shows that the expression of each gene, in each of the HCC PDOs, is repressed by omacetaxine, in a dose-dependent fashion. Data were displayed as mean ± SD from 3 independent experiments. Paired, 2-tailed Student’s *t* test was used for the statistical analysis. *P < 0.001.

To further explore the mechanism of action for omacetaxine and its influence on gene expression, 5 HCC PDO lines were treated with 100 nM omacetaxine or control, respectively. mRNA was extracted, and real-time PCR arrays were performed with a panel of 203 genes. The heatmap of gene expression for these 203 genes is shown in Figure 5A and Supplemental Table 2. A group of genes was uniformly downregulated in each of the 5 HCC PDO lines tested. This group of genes is highlighted in green in the figure. We found that Polo-like kinase 1 (PLK1), Aurora kinase B (AURKB), and E2F transcription factor 1 (E2F1) were 3 of the most suppressed genes following omacetaxine treatment (Figure 5B). PLK1 is a crucial regulator of cell cycle progression, centriole duplication, and mitosis. Recent studies showed PLK1 as a promising target for cancer treatment (33, 34). AURKB is one of the essential kinases for cell division, including activation of PLK1. AURKB promotes cancer growth and invasion and, therefore, was found to be a promising target for cancer treatment (35). E2F1 is a critical regulator of HCC proliferation/apoptosis through PIK3CA/Akt/mTOR and c-Myc/COX-2 pathways (36). We concluded that, in addition to directly inhibiting the synthesis of certain short–half-life proteins, omacetaxine also indirectly downregulates other cell division/apoptosis genes such as PLK1, AURKB, and E2F.

### Omacetaxine inhibits cancer growth and increase survival in PDX models of HCC.

**Figure 6:**
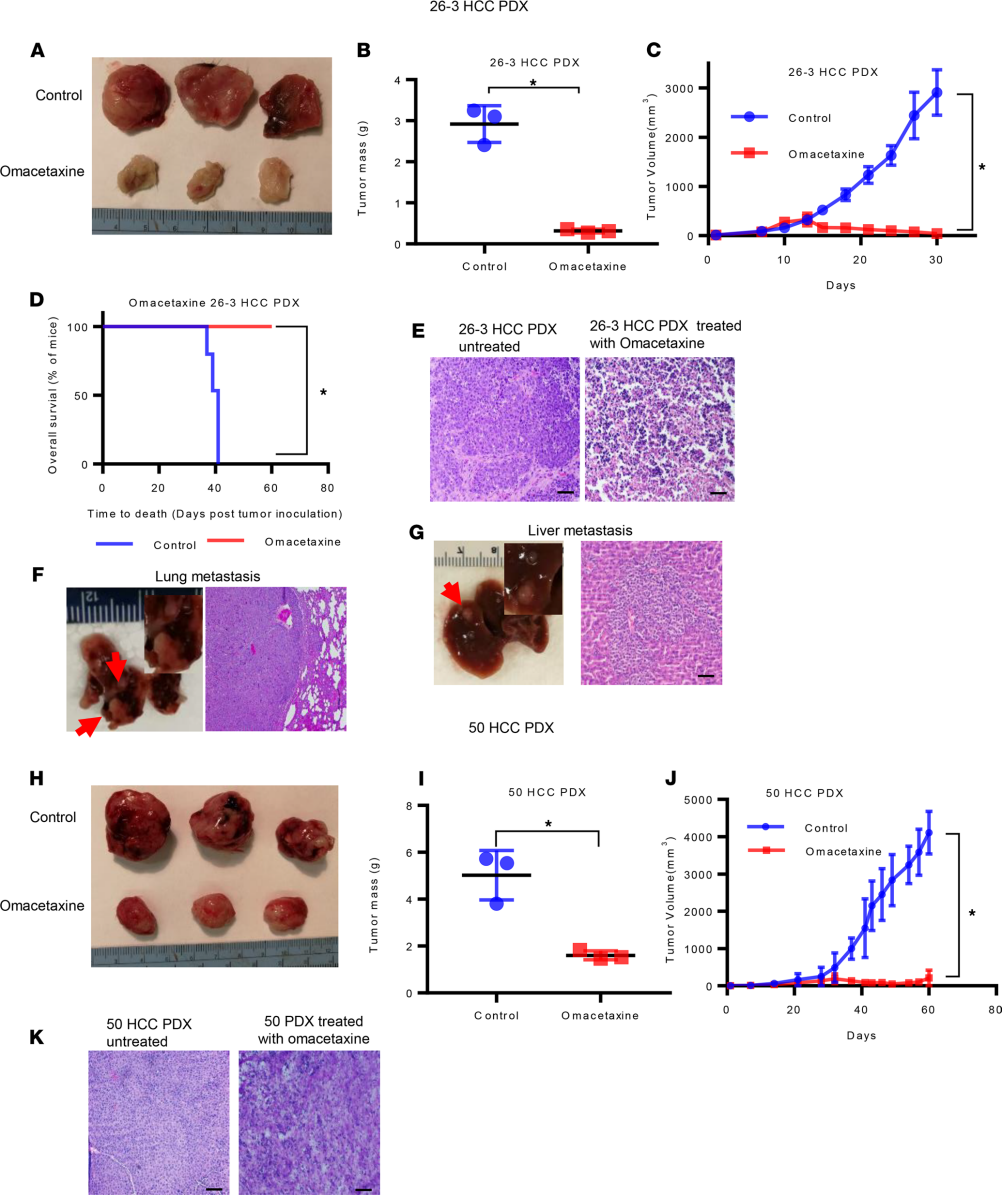
Omacetaxine inhibits HCC growth and metastasis in vivo. (**A**–**D**) The effects of omacetaxine in the 26-3 HCC PDX. (**A**) Omacetaxine dramatically inhibits the volumes of the tumors as recorded at the end of the experiment. (**B**) The difference in tumor weight between the 2 groups was significant. (**C**) There was a significant reduction in the size of the tumors treated with omacetaxine when compared with negative control–treated tumors. (**D**) Survival of mice bearing 26-3 PDX after treatment. Significance was determined with log-rank test. *P < 0.01. (**E**) H&E staining of 26-3 HCC PDX treated with omacetaxine and negative control. The untreated tumor demonstrated viable cells, while the treated tumor appears necrotic with no viable tumor cells. (**F** and **G**) Gross pictures and H&E staining of lung and intraliver metastases of 26-3 HCC PDX control group sacrificed at day 30. (**F**) Lung metastases. Gross picture shows 2 firm, white metastatic nodules (red arrows). Histology showed malignant tumor cells in the alveoli of the lung (right), similar to the primary liver tumor in 26-3 HCC PDX. (**G**) Liver metastasis. The gross picture shows a white nodule in the liver parenchyma (red arrow). The H&E image shows infiltrative neoplastic cells present in normal hepatic parenchyma. (**H**–**J**) Omacetaxine exhibits similar growth inhibition effects in the second PDX model (50 HCC PDX). (**K**) H&E staining of the original 50 HCC tissue and 50 HCC PDX treated with omacetaxine and negative control. Data were mean ± SEM from each group. Two-tailed Student’s *t* test was applied for the statistical analysis. *P < 0.001.

PDX models of human cancer are well documented to recapitulate primary tumor biology, as well as response to treatment (37, 38). To investigate if omacetaxine displays effectiveness in vivo, we utilized both the 26-3 HCC PDX (which is a more aggressive cancer with metastatic potential) and 50 HCC PDX (a less aggressive cancer). We found that i.p. omacetaxine treatment resulted in halting tumor growth in both of these HCC PDX models (Figure 6, A and H). At the end of the treatment, there was a statistically significant difference in the size of the tumors, as well as in the weight of the tumors, for each of the PDX models used (Figure 6, B, C, I, and J). As seen in Figure 6E (for 26-3 HCC PDX) and Figure 6K (for 50 HCC PDX), H&E staining of untreated versus treated tumors demonstrated that, after the treatment with omacetaxine, tumors become necrotic with no obvious viable cancer cells. In addition, we investigated if there was a survival advantage provided by the treatment with omacetaxine. PDX50 is less aggressive — mice continue to be active, to eat and function normally, in spite of very large tumors. Therefore, PDX50 is not ideal for survival determinations. PDX26-3, however, is more aggressive (mice die due to the cancer burden). A survival experiment in which PDX26-3 was utilized was designed and implemented, and it demonstrated a statistically significant increase in survival of approximately 100% (Figure 6D).

Furthermore, we also noted that the control 26-3 HCC PDX mice developed several liver and lung metastatic sites (Figure 6, F and G). Importantly, no metastatic loci were found in the 26-3 HCC PDX mice treated with omacetaxine. These data strongly argue that omacetaxine is effective in vivo in 2 PDX models of human HCC, and this fact predicts its effectiveness in patients.

### Omacetaxine inhibits cancer proliferation and increases apoptosis in vivo in PDX models of HCC.

**Figure 7:**
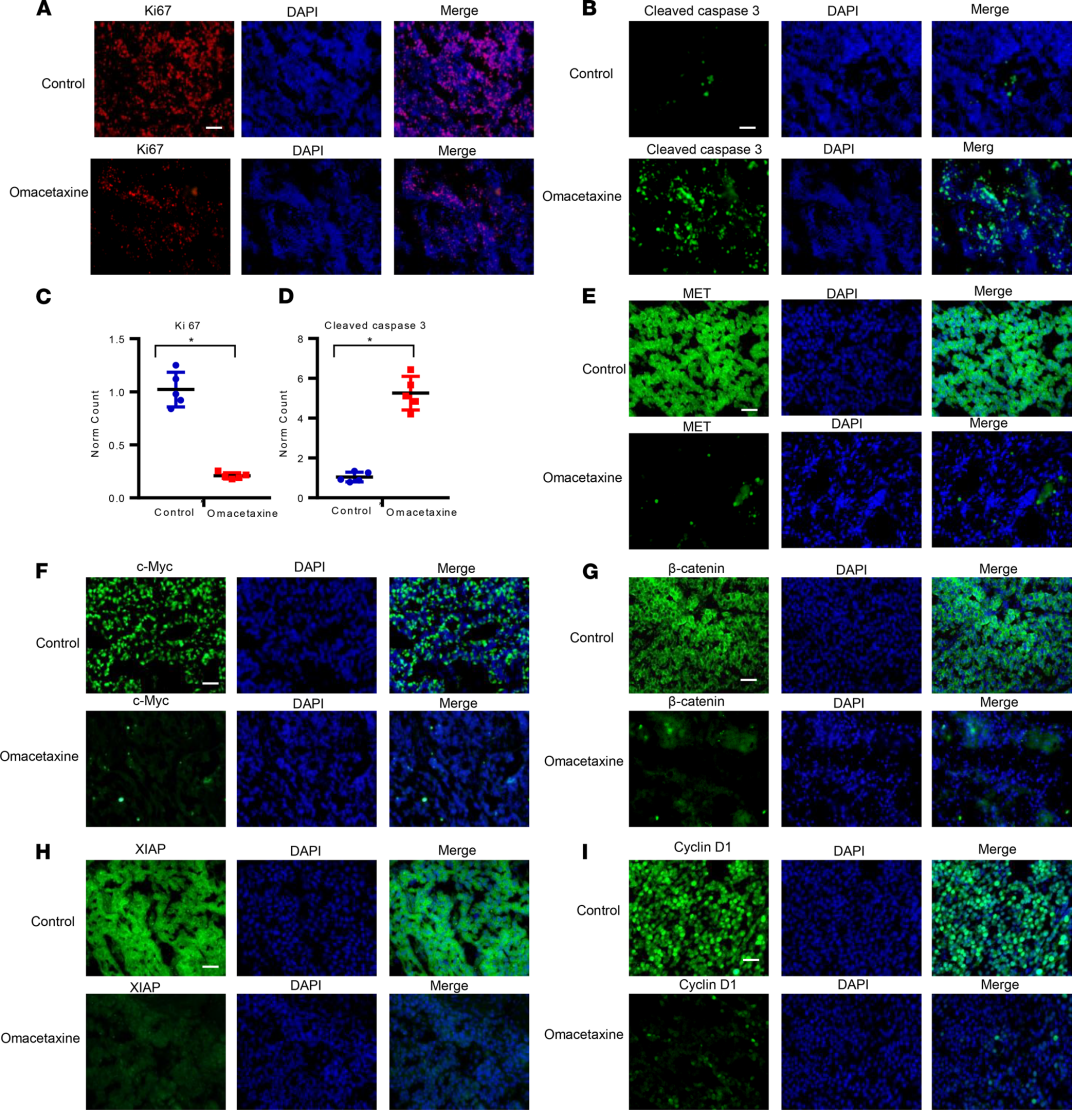
Omacetaxine inhibits cell proliferation, increases apoptosis, and suppresses oncoproteins in vivo. (**A** and **B**) Ki-67 and cleaved caspase 3 expression in the 26-3 HCC PDX model demonstrates that omacetaxine decreases proliferation and increases apoptosis in vivo. (**C** and **D**) Normalized data of Ki-67 and cleaved caspase 3 in omacetaxine treatment versus control. Scale bar: 100 μM. Data are mean ± SD from 5 independent measurements. Paired 2-tailed Student’s *t* test analysis was used for the statistical analysis. *P < 0.001. (**E**–**I**) Omacetaxine suppresses the following oncoproteins in vivo: MET, c-Myc, β-catenin, XIAP, and cyclin D1. Scale bar: 50 μM.

To verify the mechanism of omacetaxine against HCC in vivo, omacetaxine-treated HCC PDX tumors, as well as matched controls (tumors treated with the negative control), were harvested, and frozen section slides were made. We found that proliferation was less, as measured by Ki-67 protein expression (Figure 7A). Apoptosis was also increased in omacetaxine-treated cancers, as quantified by cleaved caspase 3 staining (Figure 7B). The difference in proliferation, as well as apoptosis, was statistically significant (Figure 7, C and D).

### Omacetaxine downregulates short–half-life oncoproteins in vivo in PDX models of HCC.

We have demonstrated that omacetaxine induces (a) global downregulation of protein synthesis (Figure 4, A and B) and (b) downregulation of short–half-life oncoproteins in vitro (Figure 4, C–G). The demonstration of similar findings in vivo would further argue that omacetaxine exerts its effects, at least in part, through inhibition of certain short–half-life oncoproteins. Tumor masses from HCC PDX mice treated with omacetaxine and negative control were utilized to prepare frozen sections, which were then stained for the c-Myc, MET, β-catenin, XIAP, and cyclin D1. As shown in Figure 7, E–I, omacetaxine downregulated in vivo the expression of all of these short–half-life oncoproteins.

## Discussion

Few HCC patients are diagnosed at early enough stages to be eligible for curative-intent treatments, such as surgical resection, ablation, or liver transplantation (4). Patients with advanced-stage disease have limited chemotherapy options. Subsequent to the approval of sorafenib, several additional targeted therapies (regorafenib, levantinib, cabozantinib, and ramucirumab), as well as 2 immunotherapies (nivolumab and pembrolizumab) have been approved, but the estimated 5-year survival rate for all patients with HCC remains only 18% (3). Recently, a combination of atezolizumab and bevacizumab became first-line therapy options for advanced HCC. It is likely that future research will result in better patient stratification so that the choice of drugs can be tailored to a particular patient group. Further research is needed to identify patient strata in which these drugs — as well as newer strategies, such as omacetaxine — are most effective. The clinical positioning of omacetaxine in the treatment of HCC remains to be established through clinical trials. The PDO and PDX models established in this project were based on tissues from patients with Barcelona clinic liver cancer A (BCLC-A) tumors that were amenable to surgical resection and previously untreated. Therefore, we don’t have data about the effectiveness of omacetaxine in previously treated HCC versus untreated HCC. It is likely that, at least initially, omacetaxine will be tested in HCC patients who have progressed on FDA-approved therapies. Additional considerations in terms of positioning omacetaxine stem from the design of the studies leading to its approval. At the time, there were concerns about home reconstitution of omacetaxine. Therefore, the FDA mandated that the first treatment be given in a medical facility daily for up to 14 days, with subsequent doses given by a home care nurse. These real-world challenges would similarly affect utilization of omacetaxine for HCC (39).

Successful establishment of HCC PDOs has been reported by us and others (12, 15). In addition, we have reported that medium-throughput drug screening in HCC PDO is feasible (15). Here, we bring proof that drug screening in HCC PDOs can be utilized for drug discovery or drug repurposing. In addition, in this new work, we add another element to the drug discovery and validation pipeline: PDX models. These models have been established, in a variety of cancers, as excellent platforms on which to recapitulate cancer biology, as well as on which to test drugs (37, 38). Omacetaxine is newly shown here to be effective in each of 2 PDX models and, by extension, should demonstrate efficacy in patients from whom these PDX models were derived, as well as possibly in even larger HCC cohorts. In addition to the successful identification and validation of omacetaxine as a potentially novel therapeutic agent, the current study presents further evidence that PDO/PDX models are effective tools that can be incorporated in drug screening strategies.

DNA sequencing shows strong evidence that APOB, RP1L1, and p53 mutations are driver mutations across the majority of PDO and PDX lines established and analyzed here. While PDX26 was more similar to the majority of PDO lines, PDX50 exhibited a unique mutational signature: ARID1A1 is a likely driver gene, and mutations in p53, APOB, and RP1L1 are notably absent. The mutational profiles identified across these samples argue that these PDO and PDX lines are representative for mutational profiles and driver mutations generally found in HCC patients. Furthermore, the fact that PDX50 is mutationally divergent from the majority of the HCC PDO lines analyzed in this study, and yet responds well to omacetaxine, highlights the strength of a nontargeted therapeutic. Interestingly, the same conclusion was reached in CML patients, and the FDA approval is specifically for CML patients who had been treated with targeted therapies. Some of these patients who had been treated with TKIs and developed resistance, intolerance, or insufficient response or developed progression still demonstrated a good response to omacetaxine. The fairly uniform good response to omacetaxine across all 40 HCC PDO and 2 PDX lines, coupled with a variety of mutational profiles in these PDO/PDX lines, argue that — similar to CML — omacetaxine could be positioned to HCC patients who are either intolerant to or progress while on targeted/immunotherapies.

Omacetaxine was originally found in Chinese herbal extracts from the bark of the Chinese plum yew, Cephalotaxus (40). This bark extract was known in traditional Chinese medicine to have anticancer activity. This extract was brought to the Western world and characterized in the early 1970s (41–43). Initial studies in China were performed with an unclear combination of active compounds, but they have demonstrated activity against AML and CML (41, 42). More recently, the FDA approved omacetaxine for the treatment of certain subtypes of CML (44, 45). This accelerated approval was based on 2 open-label trials in CML patients with T315I mutations or in CML patients who developed resistance or intolerance to at least 2 prior TKIs (44, 45). Other preclinical studies demonstrated efficacy of omacetaxine in pancreatic cancer cell lines and lung cancer mouse models (46, 47). The interest in omacetaxine prompted a recent phase I trial investigating the pharmacokinetics and excretion of omacetaxine in patients with advanced solid tumors (48). Nonetheless, to our knowledge, there have been no efficacy trials of omacetaxine in any solid tumors. The current study, to our knowledge, is the first to report that omacetaxine is effective in HCC.

Omacetaxine is a protein translation inhibitor (18). This drug does not appear to target a specific protein, but rather inhibits overall protein translation. Overall inhibition of protein translation predominantly affects proteins with rapid turnover and a short–half-life (18). Omacetaxine is efficacious in CML due to blockade of oncoproteins that have short half-lives and which, by default, depend on rapid protein synthesis (32, 49). In this study, we show, for the first time to our knowledge, that omacetaxine affects global protein translation in HCC cells. Furthermore, we show that several oncoproteins (c-Myc, MET, β-catenin, XIAP, and cyclin D1) decrease markedly following treatment with omacetaxine, both in PDOs in vitro and in PDX models in vivo. In addition, real-time PCR array analyses of omacetaxine-treated PDOs indicated that omacetaxine inhibits several proto-oncogenes, notably including PLK1, AURKB, and E2F1. Therefore, it is likely that the activity of omacetaxine in HCC, as in CML, is mediated by direct global inhibition of protein translation that affects predominantly short-lived oncoproteins and indirect inhibition of other oncogenes. Lastly, a key aspect of any future drug in this class of global protein inhibitors appears to be its transient activity, which would avoid affecting proteins with longer half-lives (18). Further efforts to position omacetaxine for HCC treatment should take into consideration this need for its transient utilization.

Of great translational importance, omacetaxine induces toxicity in HCC cells at much lower doses that in normal human liver cells. In our study, the dose required to induce toxicity in human normal liver cells is 5.5- to 17-fold higher than the dose required to induce toxicity across all 40 HCC PDO lines. Future studies are required to establish a dosing regimen for HCC, as well as the positioning of omacetaxine in the therapeutic arsenal for HCC.

## Methods

### HCC patients and samples.

Fresh human HCC tissue was obtained from procedures (surgery or needle biopsy) performed at the Johns Hopkins Hospital (JHH).

### Establishment of HCC PDOs and normal liver PDOs.

HCC PDOs and normal human liver organoids, as well as mouse normal liver organoids, were established as described (15). Details are included in Supplemental Data.

### PDX establishment.

In this study, we established 2 different HCC PDX models. Five- to 6-week-old female NSG (The Jackson Laboratory) were utilized. Details are included in Supplemental Data.

### DNA sequencing.

Fifteen of the 40 PDO and 2 PDX lines established in our lab had DNA available for exome sequencing. The DNA was extracted using QiAamp DNA min kit DNA (Qiagen); DNA sequencing was performed by Novogene. Normalization and data quality control were performed with Trim Galore! (50) and FastQC (51). Reads were aligned to GRCh38 with hisat2 (52), reprocessed with Opossum (53) and Platypus (54), and annotated with ANNOVAR (55), with information from the refGene (56) and COSMIC (57) databases. Custom Python scripts were used to collate the ANNOVAR results.

For variants within known HCC driver genes, we performed an additional level of analysis to distinguish between driver mutations and passenger mutations without functional impact. We used CHASMplus (58) to predict the likelihood that any given variant is a driver rather than a passenger mutation. OpenCRAVAT (59) was used to annotate exonic variants with predicted driver scores from CHASMplus’s liver cancer (LIHC) model. CHASMplus scores range from 0 (least likely to be a driver) to 1 (most likely to be a driver mutation) and are tabulated in Supplemental Table 1.

Counts of exonic variants within driver genes (60–62) were tabulated for each sample sequenced and included in the analysis (Supplemental Table 1). Of the 1305 variants found in these genes, refGene identified 300 as exonic. CHASMplus gave 108 of those a nonzero score as drivers. CHASMplus scores were interpreted as nonfunctional (zero score) or possibly functional (score above zero), and then whether the CHASMplus score was above or below 0.1. Green indicates that 2 or more of the variants for a particular combination of patient and gene scored above 0.1. Orange indicates that the sample had 1 mutation that scored above 0.1 for a given gene. For samples that had no variants that scored over 0.1, yellow indicates that 2 or more variants were scored greater than zero but lower than 0.1, and gray indicates that 1 variant was scored greater than zero but lower than 0.1.

### Drug screening in HCC PDOs in vitro.

A panel of 129 FDA-approved anticancer drugs obtained from the NCI was utilized for drug screening on HCC PDOs, as described (15). Details regarding generating dose-effect IC_50_ calculation curves are included in Supplemental Data.

### Omacetaxine treatment.

Six HCC PDX26 mice and 6 PDX50 mice were each randomized to 2 groups. The treatment started at day 7 after implantation. At that point, the size of the PDX26 tumors was 8.54 ± 2.181 mm^3^. The size of the PDX50 tumors was 6.06 ± 1.71 mm^3^. Mice were treated with either vehicle or omacetaxine at a dose of 0.5 mg/kg via i.p. injection daily until day 30 (for HCC PDX26) or day 45 (for PDX50). S.c. tumor volumes were measured with a Vernier caliper twice a week. Animals were then sacrificed, and tumor weights were recorded. The presence of lung and liver metastases was verified by histology for both PDX models. For survival curves experiment, another 8 HCC PDX26 mice were randomized into 2 groups (*n* = 4 mice per group). Mice were treated from day 7 after the tumor was implanted until the mouse died. Further details of materials and methods are in Supplemental Data.

### Statistics.

All graphs were generated using Microsoft Excel, R software, and GraphPad Prism 7.0 (GraphPad). Data were presented as mean ± SD or mean ± SEM as specified in figure legends. The biological replicates are shown in each figure. Statistical significance with the *P* value was determined with a 2-tailed Student’s *t* test or 1-way ANOVA. A Kaplan–Meyer method and log-rank test was used for xenograft survival curve analysis. A 2-sided *P* < 0.05 or *P* < 0.001 was considered statistically significant.

### Study approval.

Patients were consented under an approved IRB from Johns Hopkins University School of Medicine. The diagnosis of HCC was confirmed by a liver pathologist at JHH. All mouse procedures were performed with approval from the Animal Care and Use Committee (ACUC) at Johns Hopkins University.

## Author contributions

LL, JSB, and FMS designed experiments. LL, GH, HH, and PD performed experiments. LL, GH, MGL, KO, JSB, and FMS analyzed data. MJW, JH, BP, RB, WRB, RNW, AMC, CLW, CG, SK, NSA, and MY provided patient-derived specimens and clinical correlations. LL, MGL, JBS, and FMS wrote the manuscript, MJW, BP, CG, SJM, LME, JSB, and FMS revised the manuscript. All authors approved the manuscript.

## Supplementary Material

Supplemental data

## Figures and Tables

**Table 1:**
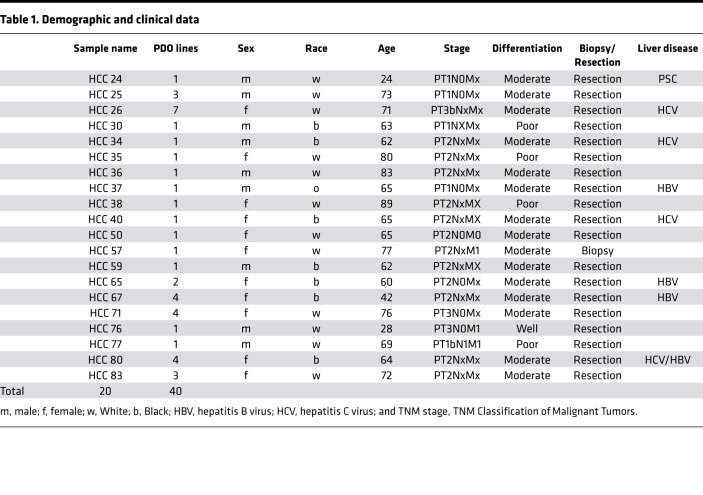
Demographic and clinical data

## Version 1 05/18/2021

In-Press Preview

## Version 2 06/22/2021

Electronic publication
