# Supplementary material for: Protein synthesis inhibitor omacetaxine is effective against hepatocellular carcinoma
Source: JCI Insight. 2021 Jun 22;6(12):e138197. doi: 10.1172/jci.insight.138197 (PMC8262474; doi:10.1172/jci.insight.138197)
Supplement: Supplemental data [file jciinsight-6-138197-s081.pdf]

Supplementary Data

Establishment of PDOs from human HCC specimen

HCC tissue was harvested under sterile condition from fresh surgical specimens or from core needle biopsies. The tissue was collected in PBS and transported to the laboratory on ice. The tissue was then washed three times with cold DMEM plus 1% Penicillin/streptomycin, minced into small pieces, and washed twice to remove debris. Subsequently, the tissue was subjected to dissociation buffer (DMEM with DNase (0.1 mg/ml) as well as collagenase I (2mg/ ml)). The cells were dissociated at 37ºC in a water bath, and were shaken every 10-20 min. Dissociation was stopped with DMEM with 10% FBS. Next, cells were filtered with a 100 µM cell strainer. Finally, cells were mixed with growth factor reduced matrigel (Cat#: 356231, Corning MA) in 24 well plate and placed in a cell culture incubator for 15 min. After matrigel solidification, warm medium was added to the wells. The HCC PDO growth medium was made as described previously [15]. Medium was changed every 3-4 days, and PDOs were passaged every 1-2 weeks. **Table 1** displays demographic and other relevant clinical data.

HCC PDX model establishment

HCC PDX26 was derived from a diffuse infiltrative HCC. This PDX model is an aggressive cirrhotomimetic HCC with early metastatic ability. To establish HCC PDX26, $2X10^6$ 26-3-HCC PDO cancer cells mixed with matrigel (1:1) were subcutaneously injected into the right flank of NSG mice (5-6-week old, male, Jackson Laboratory). Once established, the PDX tumor tissue was cut into cubes (1 mm^3), and subcutaneously implanted into the right flank of NSG mice for passage. In NSG mice, HCC PDX26 tumors usually metastasize within 30 days to distant sites, such as the lung. HCC PDX50 is a moderately differentiated nodular HCC with low metastasis ability. To establish HCC PDX50, fresh patient derived HCC tissue was harvested, washed with cold PBS, cut into small cubes (1 mm^3) in a sterile hood, and implanted into the right flank of NSG mice.

IC50 curve generation

For each PDO line, approximately 2,000 cells were mixed with matrigel and plated in triplicate in 96 well plates. 100 uL medium was added per well. After 48 hours, the medium was changed to fresh medium containing drugs at different concentrations. Cell viability was determined with a CellTiter Glo luminescence assay (Cat#: G7571, Promega, WI). Data were collected on a Perkin-Elmer EnVision plate reader. IC50 values were determined by a nonlinear best-fit method using GraphPad (GraphPad, San Diego, CA).

Immunofluorescence

Fresh human HCC, PDO and mouse PDX tissues were frozen in OCT at -20 ºC immediately after being harvested. Slides of 10 uM sections were utilized for immunohistochemistry or immunofluorescence staining as described before [15]. H&E staining was performed following the standard protocol. Pictures were recorded with Zeiss Axio Observer Inverted Microscope as described previously [15].

Apoptosis assay

PDOs were treated with 0.1% DMSO vehicle or omacetaxine at different concentrations (0.3 uM and 0.7 uM) for 4 days. PDO cells were collected and dissociated into single cells using TrypLE Express Enzyme and DNase. Apoptosis was determined by using Annexin V Apoptosis Kit (Cat#: 88-8005-72, BD Biosciences) and analyzed by flow cytometry (Sony Cell Sorter SH800).

DNA synthesis assay

Cells allowed to incorporate BrdU were analyzed with the APC BrdU flow kit (BD Biosciences), following the manufacturer's protocol. Briefly, omacetaxine at different concentrations was utilized to treat 6 different HCC PDOs. Cells were stained with APC-conjugated anti BrdU antibody, then subjected to flow analysis to assess the proportion of cells in S-phase. Data were analyzed using the FlowJo software (BD Frankin Lakes, NJ)

Cell cycle analysis

Six HCC patients PDOs were treated with omacetaxine at at 0 uM, 0.15 uM, 0.3 uM and 0.7 uM for 4 days. Briefly, organoids were digested into single cells. Single cells were washed with cold PBS and suspended into 100 ul cold PBS. Single cells were fixed in 3 ml pre chilled 70% ethanol with vortexing. After 30 min, cells were washed with cold PBS twice, then treated with RNase 100 ug/ml at 37 degree for 1 hour. 200 ul propidium iodide (PI) was utilized to stain the DNA. The DNA content was analysis with flow cytometry. Data was analyzed with the FlowJo software (BD, Frankin Lakes, NJ).

Flow cytometry analysis of protein expression

PDOs that were treated with omacetaxine were collected and digested into single cells, then washed with cold PBS twice and fixed with flow cytometry fixing buffer. Intracellular protein staining was performed following the standard protocols from the manufacturer (BD Biosciences, San Diego, CA). The protein expression was analyzed with the SONY SH800 Cell sorter machine.

RNA extraction and real time PCR array

5 PDO lines were treated with 100 nM Omacetaxine and control (0.1% DMSO) for 4 days, cell were collected and RNA were extracted with Qiagen RNEasy mini kit. CDNA were synthase using Revert Aid First Strand cDNA Synthesis Kit (Cat: K1622, ThermoFisher Scientific).cDNA were applied to a human liver cancer gene, human Cancer drug Targets/resistance gene array (Qiagen), Reactions were performed using a QuantStudio 3 PCR machine. The ½^ddct method was used with normalization of data to the mean to the CT value of 3 independent housekeeping genes.

Global protein synthesis assay

PDOs were seed in 24 well plate. After 24 hours the medium was replaced with free aliquots containing EZclick protein label and omacetaxine as well as positive control Cycloheximide. Cells without EZclick protein label served as negative control. Cells were incubated for 24 hours. Cells were harvested fixed and permeabilized. The EZClick protein reaction cocktail was then added and incubated for 30 min (EZClick^{TM}, Global Protein Synthesis Assay (Cat#: K459-100, BioVision, CA).. Flow cytometry was utilized to determine global protein inhibition.

Antibody

The following antibodies were utilized for IF and flow cytometry: Cleaved Caspase-3 (Cat#: Asp175, Cell Signaling), β-catenin (Cat#:8480, Cell Signaling), MET (Cat#:8198, Cell Signaling), XIAP (Cat#: PA5-29253, Invitrogen), XIAP (Cat#:ab28151, Abcam), Cyclin D1 (Cat#: PA5-85257, Invitrogen), Cyclin D1 (Cat#: ab16663, Abcam), Ki67 (cat#: 550809, BD Biosciences), LGR5 (Cat#: AP2745, Abgent), EPCAM (Cat#: 2929, Cell Signaling), CK19 (Cat#: MAB3238, Millipore), HepPar1 (Cat#: 264R-1, Sigma Aldrich), AFP (Cat# PA516658, ThermoFisher Scientific), AlexaFluor 594 or 488 secondary antibodies (Life Technologies). For live/dead cell staining we utilized the Cell Tracker Green/ Ethidium homodimer-1 (Cat#: C7025 and Cat#: E1169, Life Technologies).

Supplementary Table 1: Determination of driver mutations from DNA-sequencing.

	ACVR2A	AHCTF1	ALB	APOB	ARID1A	ARID2	ATM	AXIN1	AZIN1	BAP1	BRAF	CCND1	CREB3L3	CTNNB1	EEF1A1	ERRFI1	GPATCH4	HIST1H1C	HRAS	IGF2	IL6ST	JAK1	KEAP1	KRAS	LZTR1	MET	MYC	NCOR1	NFE2L2	NRAS	PIK3CA	PTEN	RB1	RP1L1	RPS6KA3	TERT	TP53	TSC1	TSC2	VEGFA
HCC 26-3			1	5		1		1				1			2		1		1			1	1		1	2								14	1	2	1			
HCC 26-3 PDX		1		6	8		1	4	2			1	1		1		1	1	1		2					3		3						19		1	1	2		
HCC 26-5			1	5		1		1				1			2		1		1			1	1		1	2								17	1	2	1			
HCC 26-7			1	5		1		1			1	1			3		1		1			1	1		1	2								16	1	2	1			
HCC 30	1	4	1	5				4		1		1	2			1	1	1	1	1		5	1			2					1			17	1	1	1	2	3	1
HCC 34-4		2	1	3			2	1				1			2	1	1	1	1			1	1		1	3	1				1			17			1		3	
HCC 36-3	1	4	1	5	1			4		1		1	2			1	1	1	1	1		5	1			2					1			15	1	1	1	1	3	1
HCC 40	1	2	1	7		1	1	2	1			1					1	2	1	1	3	2	1		3	1		1						11	1		2	1	3	
HCC 50 PDX	3				31	5			7		6													3				11		2	2	12			3					2
HCC 59	1	2	1	5			3	2			1	2			1	2	1	1	1		3	2	1			2	1				1			18	1			2	1	
HCC 65-2	1	1	1	6			1	1	1			1			1		1	2			2	2	1		1	1								14		1	1			
HCC 67-1			1	7	1	1	3	2	1	3					2		1	1			2	2	1		1	2								16	1	2	1	1	2	
HCC 67-2			1	7	1	1	3	2	1	3					2		1	1			4	2	1	1	1	2								16	1	2	1	1	2	
HCC 71-1	1		1	6				3									1	2	1				1	1	1	4						1		15		1				1
HCC 77	1			6	1			3			1	1			1		1	2	1						1	1	1							11		2	1			
HCC 80-4		6	1	6			2	3				1					1	3		1	3	2	1		1			2			1			12	1	1			2	
HCC 83-3	1		1	4				3	1			1	1	1			1	1	1			3			1	1								17		1	1		3	

Key

More than one ranked 0.1 or higher

One ranked 0.1 or higher

more than one ranked lower than 0.1, no additional coloring if overlaps top two categories

one lower than 0.1, no additional coloring if overlaps with other two categories

Supplementary Table 2. Gene list from top to bottom for Fig. 5A

	Gene list for heatmap	
1	CDH1	
2	HDAC1	
3	OPCML	
4	CDH13	
5	MTOR	
6	ATF2	

7	AKT2	
8	CDC25A	
9	HIF1A	
10	HDAC11	
11	MDM4	
12	PLK4	
13	PDGFRA	
14	PARP2	
15	HGF	
16	ABCB1	
17	CXCR4	
18	PPARA	
19	ESR1	
20	HDAC2	
21	PGR	
22	BID	
23	DAB2IP	
24	NFKBIE	
25	HDAC3	
26	DLC1	
27	FLT1	
28	PRKCE	
29	NAT2	
30	CTSS	
31	CTSL1	
32	PIK3C2A	
33	PARP1	
34	CYP2B6	
35	ABCG	
36	PDGFRB	
37	EGF	
38	RELN	
39	FIGF	
40	SOCS1	
41	HHIP	
42	CDK7	
43	CFLAR	
44	PIN1	
45	ERBB2	
46	NFKB1	
47	XPC	

48	XPA	
49	ERBB3	
50	MVP	
51	CYP3A4	
52	CYP2C19	
53	PPARD	
54	IGF1	
55	CYP1A1	
56	CYP2C8	
57	CDK1	
58	UGCG	
59	ESR2	
60	NFKBIB	
61	CDKN1A	
62	CYP1A2	
63	GRB2	
64	MTDH	
65	HSP90AA1	
66	AHR	
67	PTEN	
68	RAC1	
69	PYCARD	
70	TNKS	
71	RHOE	
72	TGFA	
73	YAP1	
74	MCL1	
75	XIAP	
76	TCF4	
77	IRS1	
78	HDAC4	
79	RHOA	
80	SMAD4	
81	PRKCA	
82	TGFB1	
83	CDK9	
84	MDM2	
85	FAS	
86	ADAM	
87	TNFRSF11A	
88	EP300	

89	TXN	
90	PLK2	
91	MSH3	
92	NRAS	
93	PTK2	
94	CDK5	
95	FGF2	
96	CDKN1B	
97	BAX	
98	ATM	
99	ELK1	
100	CLPTM1L	
101	GSTP1	
102	CDK4	
103	TP53	
104	TOP1	
105	ARNT	
106	BCL2L1	
107	ERBB4	
108	TERT	
109	PIK3C3	
110	PIK3CA	
111	AKT1	
112	TPMT	
113	ABCC	
114	CYP3A5	
115	TNFSF10	
116	ERCC3	
117	CDK2	
118	CDK8	
119	FADD	
120	CTNNB1	
121	FHIT	
122	HDAC6	
123	CYP2C9	
124	ITGB1	
125	PRKCB	
126	PARP4	
127	TGFBR2	
128	MYC	
129	CDKN2A	

130	SOD1	
131	IGF2R	
132	ABCC	
133	HDAC7	
134	ABCC	
135	RARG	
136	CYP2D6	
137	VEGF	
138	GSK3A	
139	NFKBIE	
140	RXRB	
141	MET	
142	TOP2B	
143	RB1	
144	RUNX3	
145	PDL1	
146	AURK	
147	SFRP2	
148	FOS	
149	BRCA	
150	BRCA	
151	BLMH	
152	AURK	
153	CASP8	
154	HDAC8	
155	PRKCD	
156	KRAS	
157	LEF1	
158	WT1	
159	TXNRD1	
160	ANGPT2	
161	AP1S1	
162	IGF1R	
163	PPARG	
164	FZD7	
165	RASSF1	
166	NFKB1	
167	RARB	
168	SMAD7	
169	EGFR	
170	NTN3	

171	CDKN2D	
172	CYP2E1	
173	RELB	
174	RARA	
175	AR	
176	RXRA	
177	EPHX1	
178	TNFRSF10B	
179	CCNE1	
180	MSH2	
181	CCND1	
182	CTSB	
183	CTSD	
184	HSP90B1	
185	BCL2	
186	CCND2	
187	NFKBIB	
188	DHFR	
189	STAT3	
190	SULT1E1	
191	PLK1	
192	AURKB	
193	E2F1	
194	APC	
195	CCL5	
196	FLT4	
197	KDR	
198	KIT	
199	BIRC4	
200	SOCS3	
201	TLR4	
202	ABCC	
203	TOP2A	

Supplementary Figures

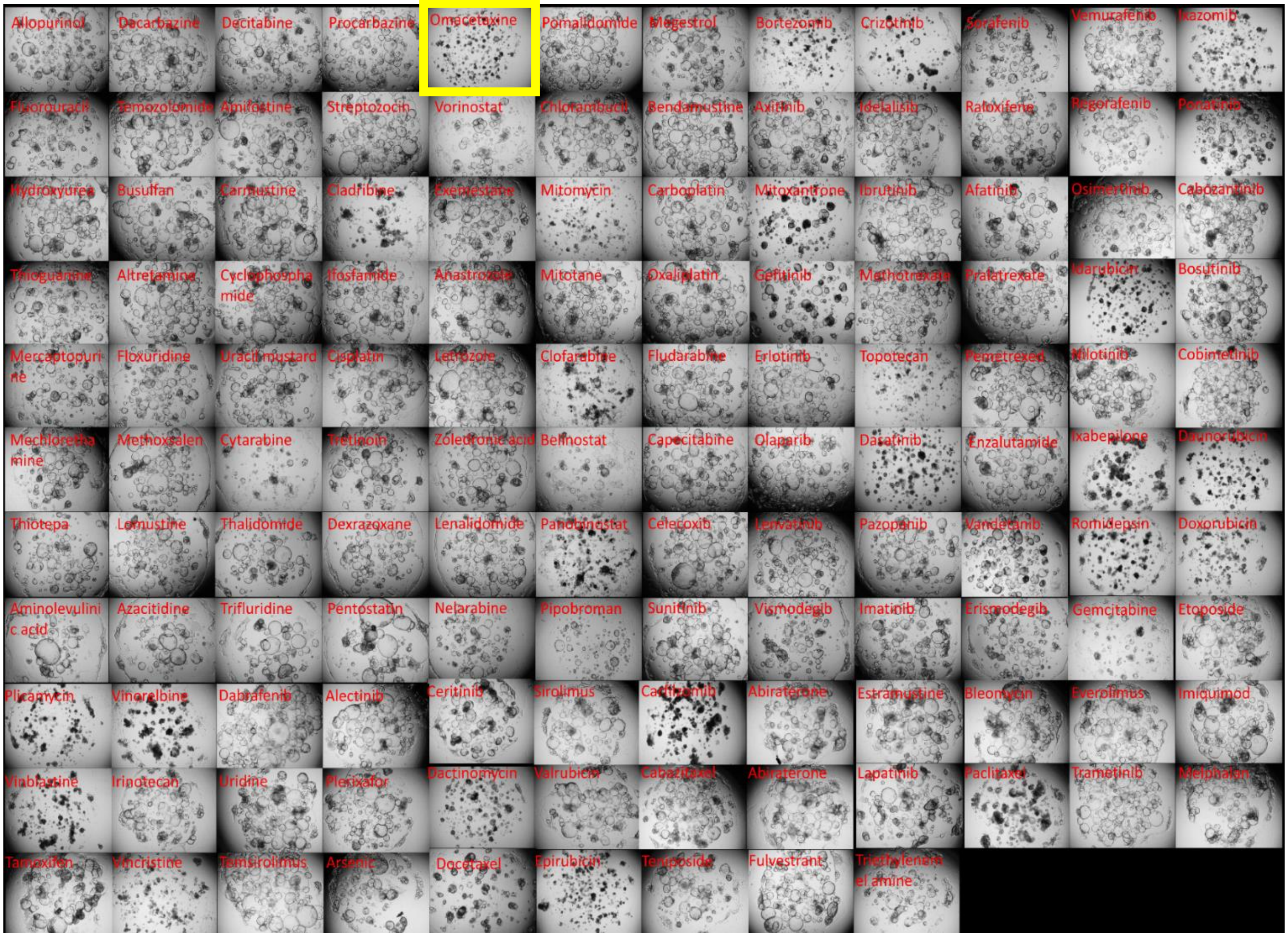

Supplementary Fig. 1. Bright field microscopy depicting efficacy of each of 129 drugs on an HCC PDO line.

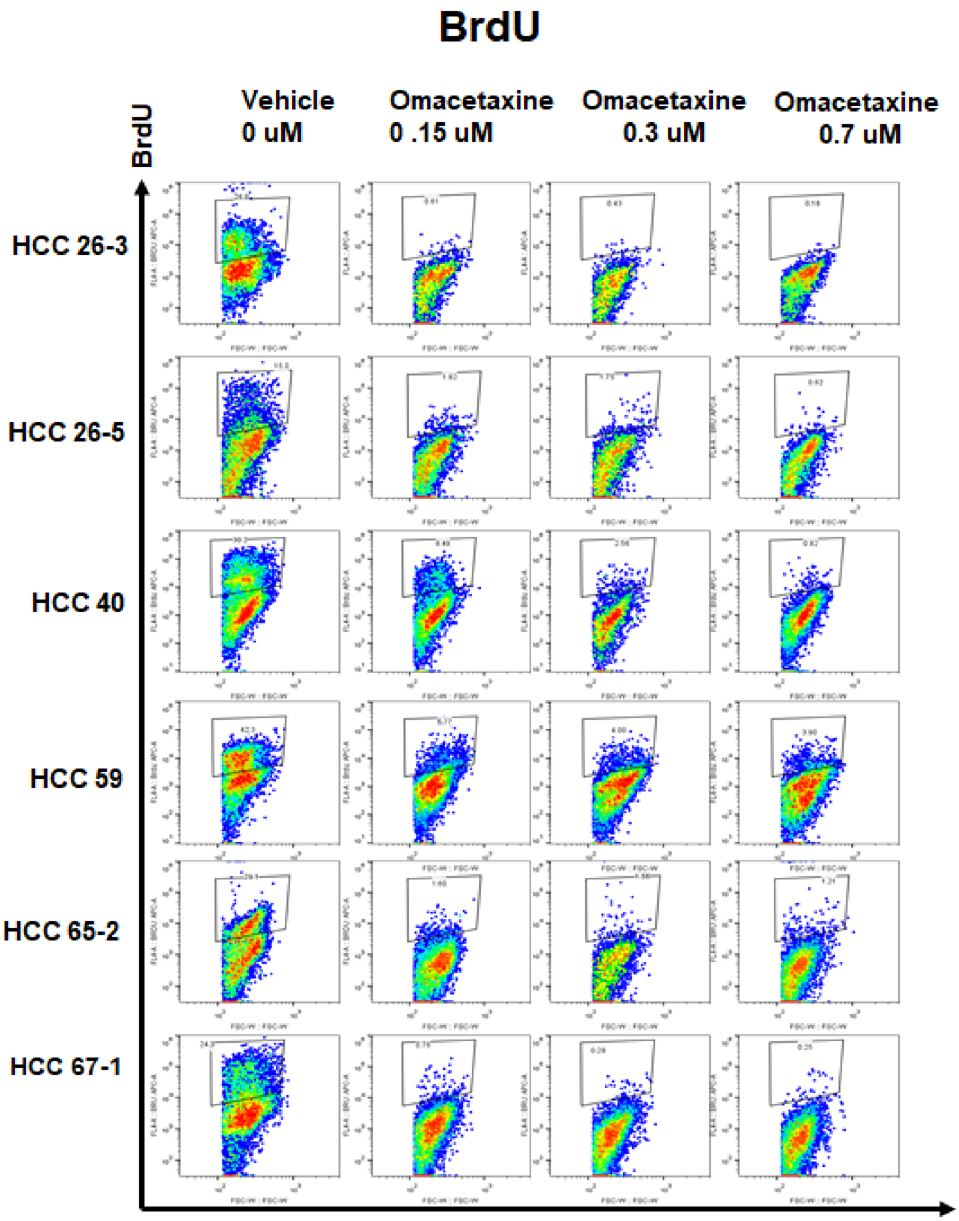

Supplementary Fig. 2. Flow cytometry gating plots data for BrdU.

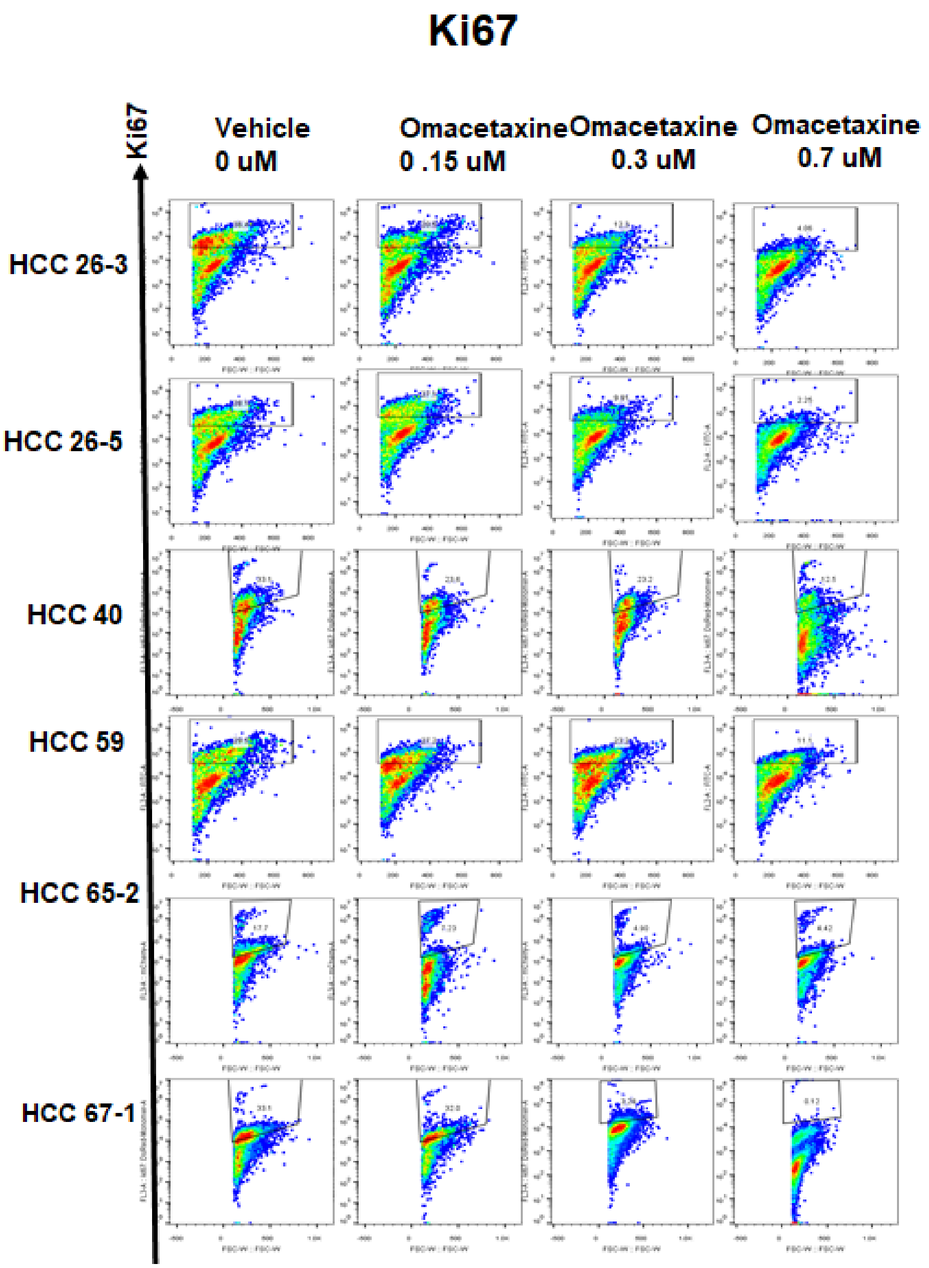

Supplementary Fig. 3. Flow cytometry gating plots data for Ki67 staining

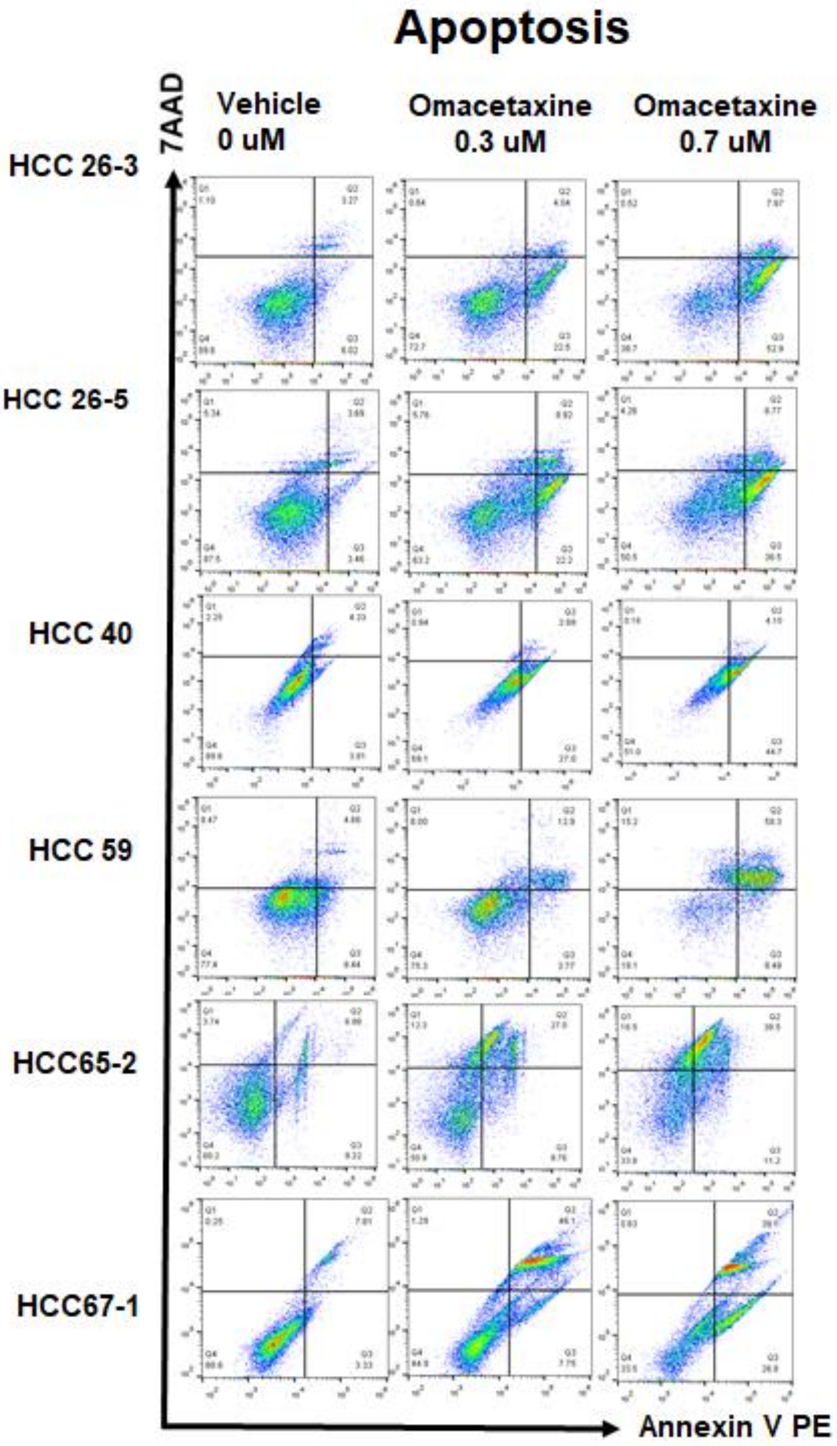

Supplementary Fig. 4. . Annexin V/7AAD. X - axis - Annexin V staining, Y - axis - 7AAD staining.

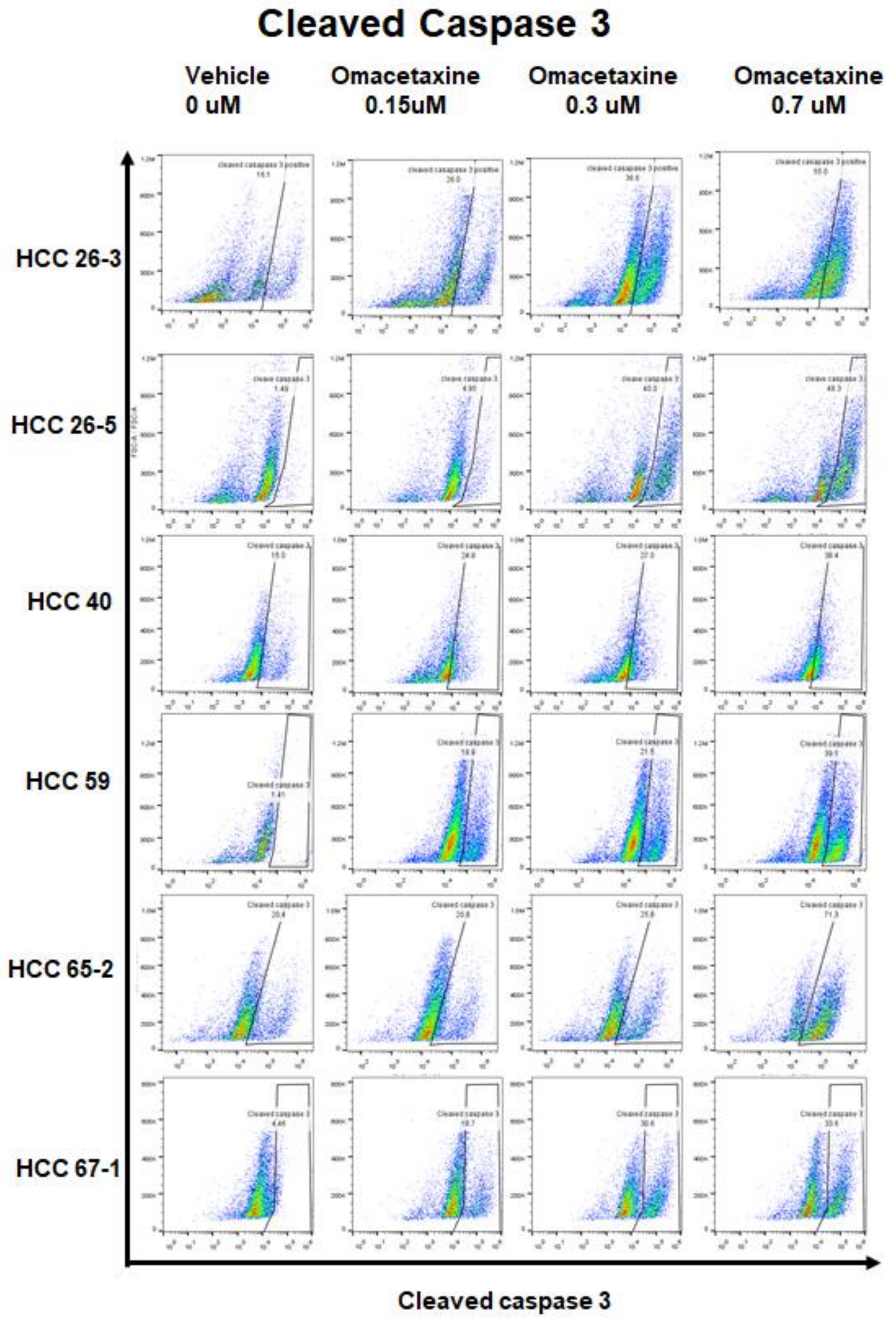

Supplementary Fig. 5. Cleaved caspase 3 expression in 6 HCC PDOs.

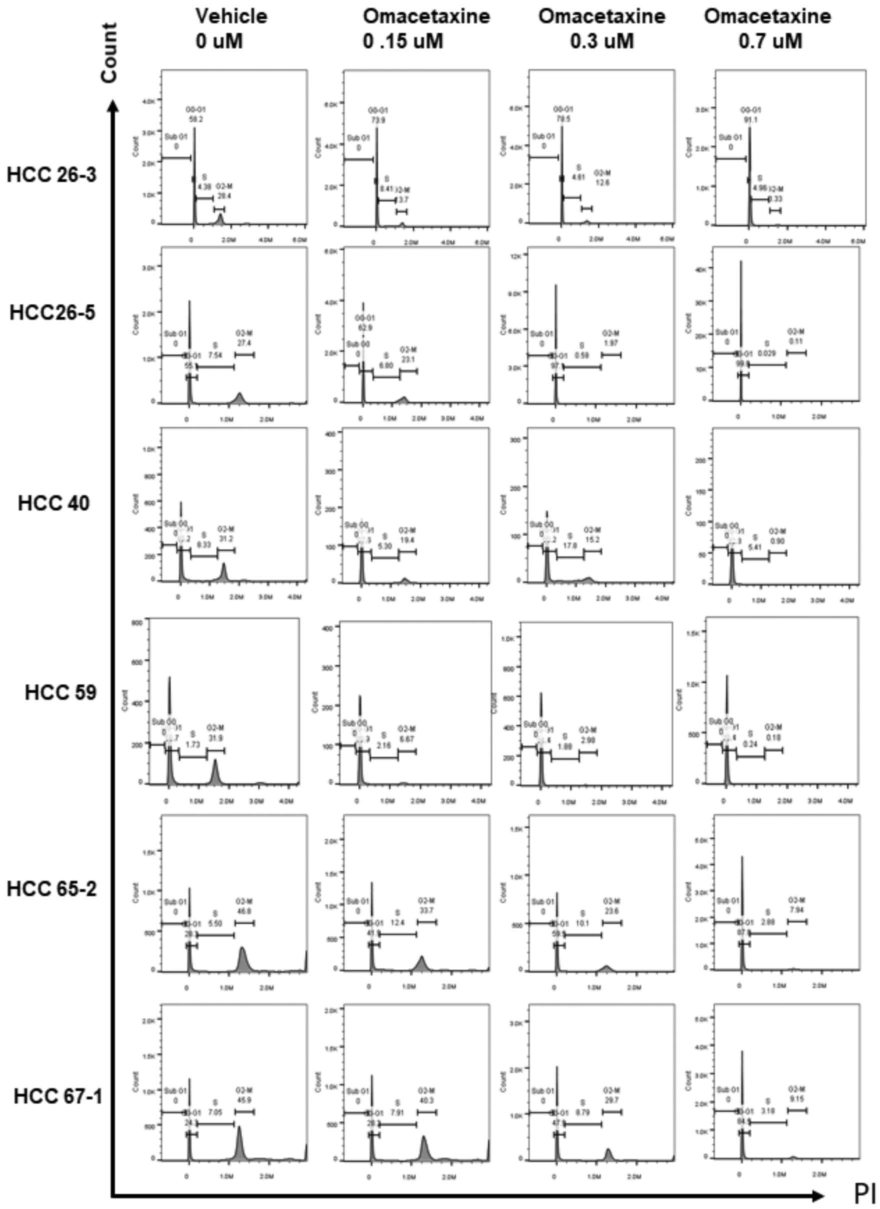

Supplementary Fig. 6. Flow cytometry Histograms of Cell cycle PI staining in 6 HCC PDOs.

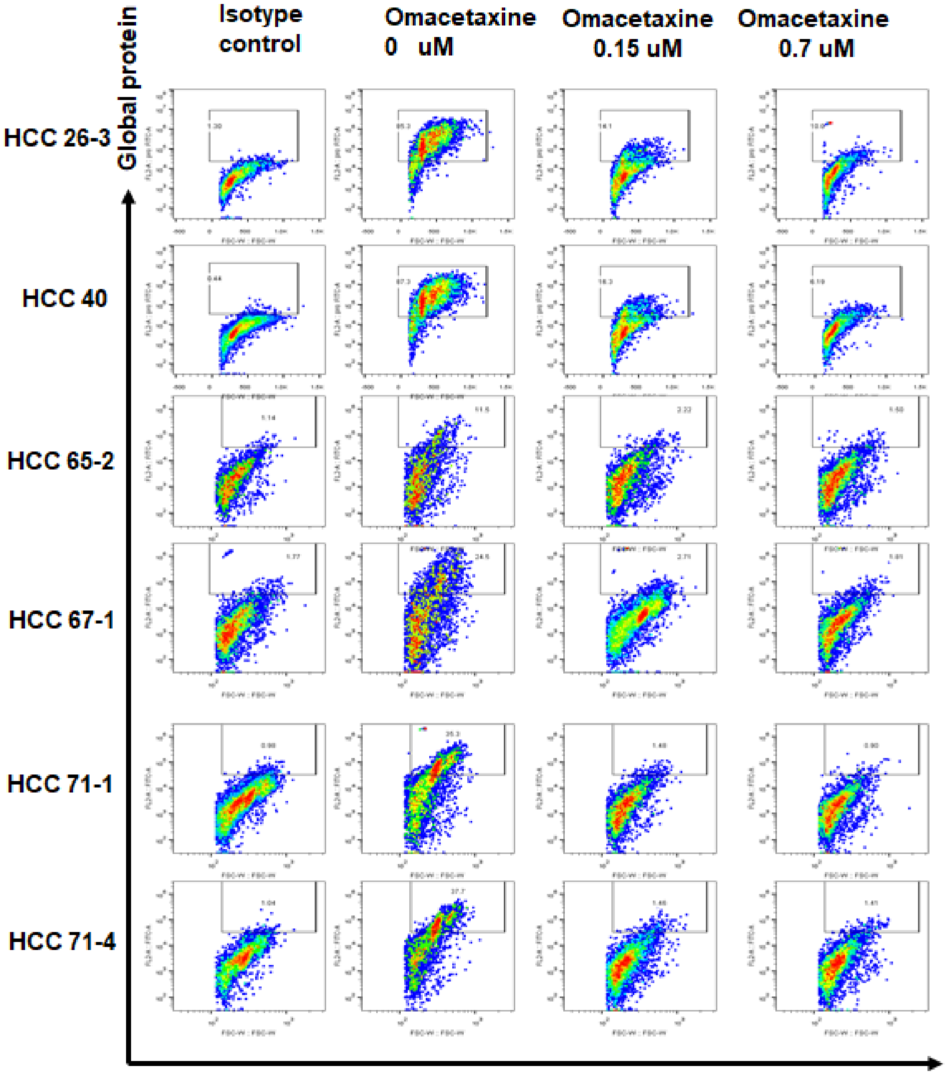

Supplementary Fig. 7. Flow cytometry gating plots data for global protein analysis.

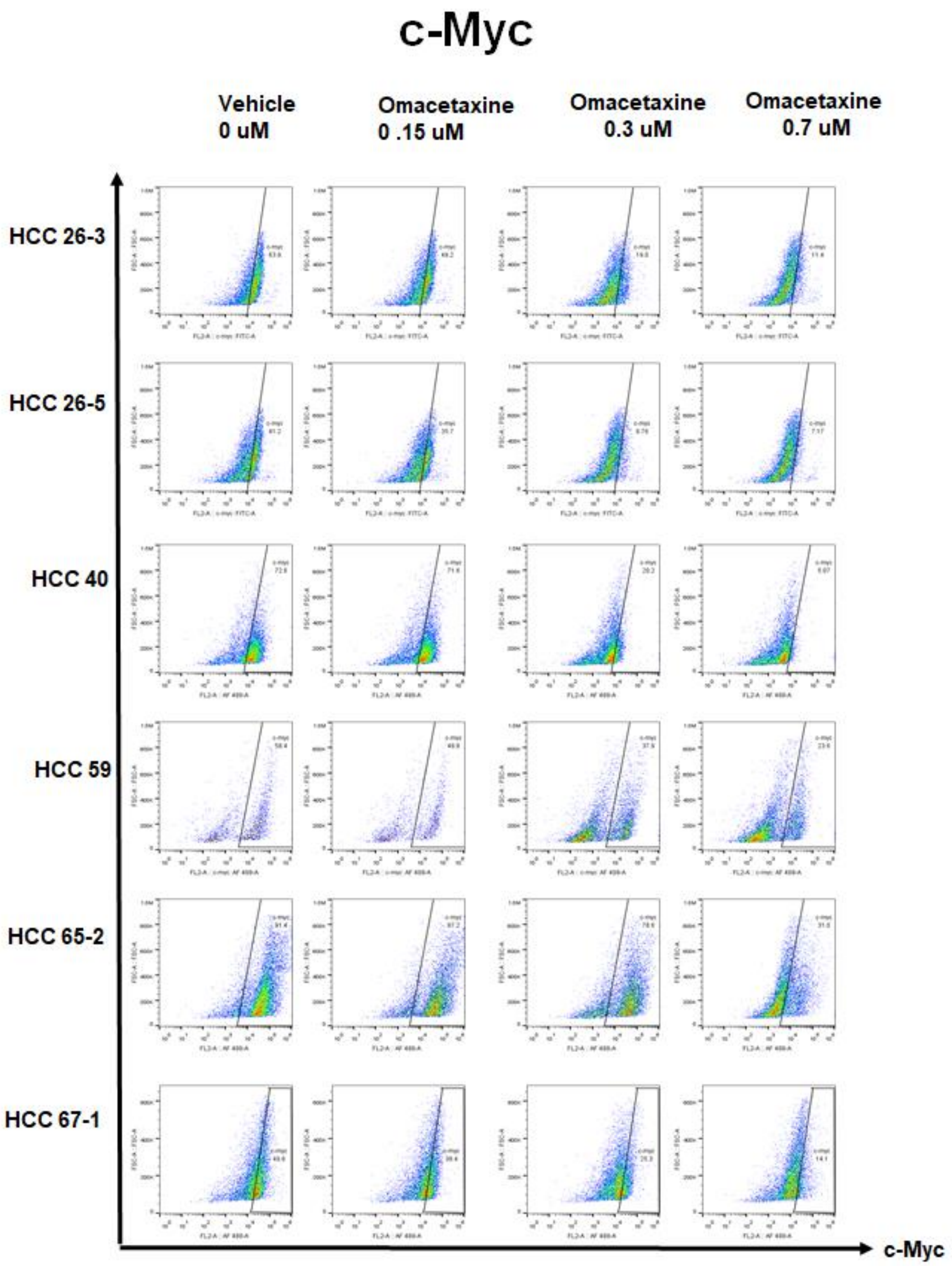

Supplementary Fig. 8. Flow cytometry gating plots data for c-Myc in 6 HCC PDOs.

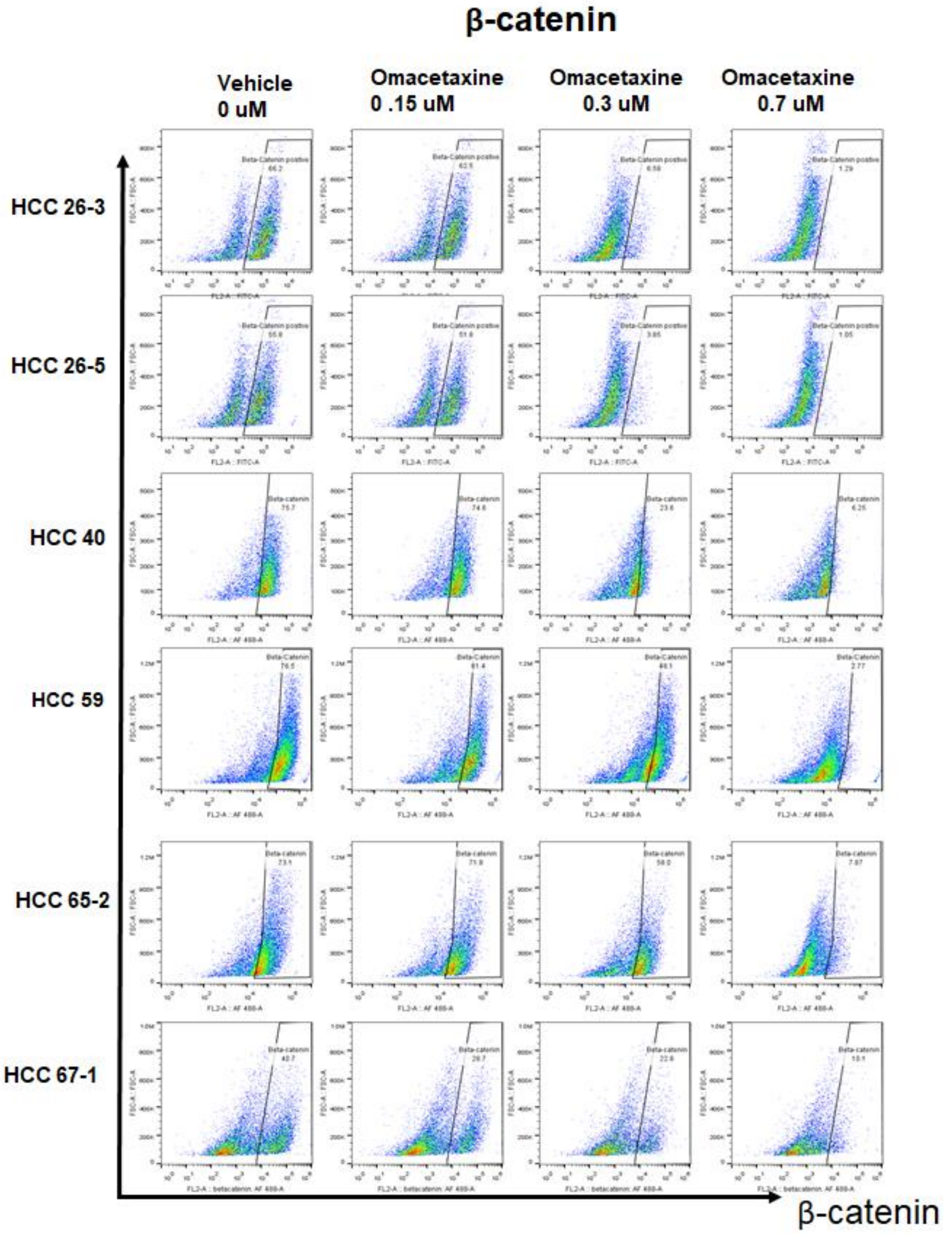

Supplementary Fig. 9. Flow cytometry gating plots data for β-catenin in 6 HCC PDOs.

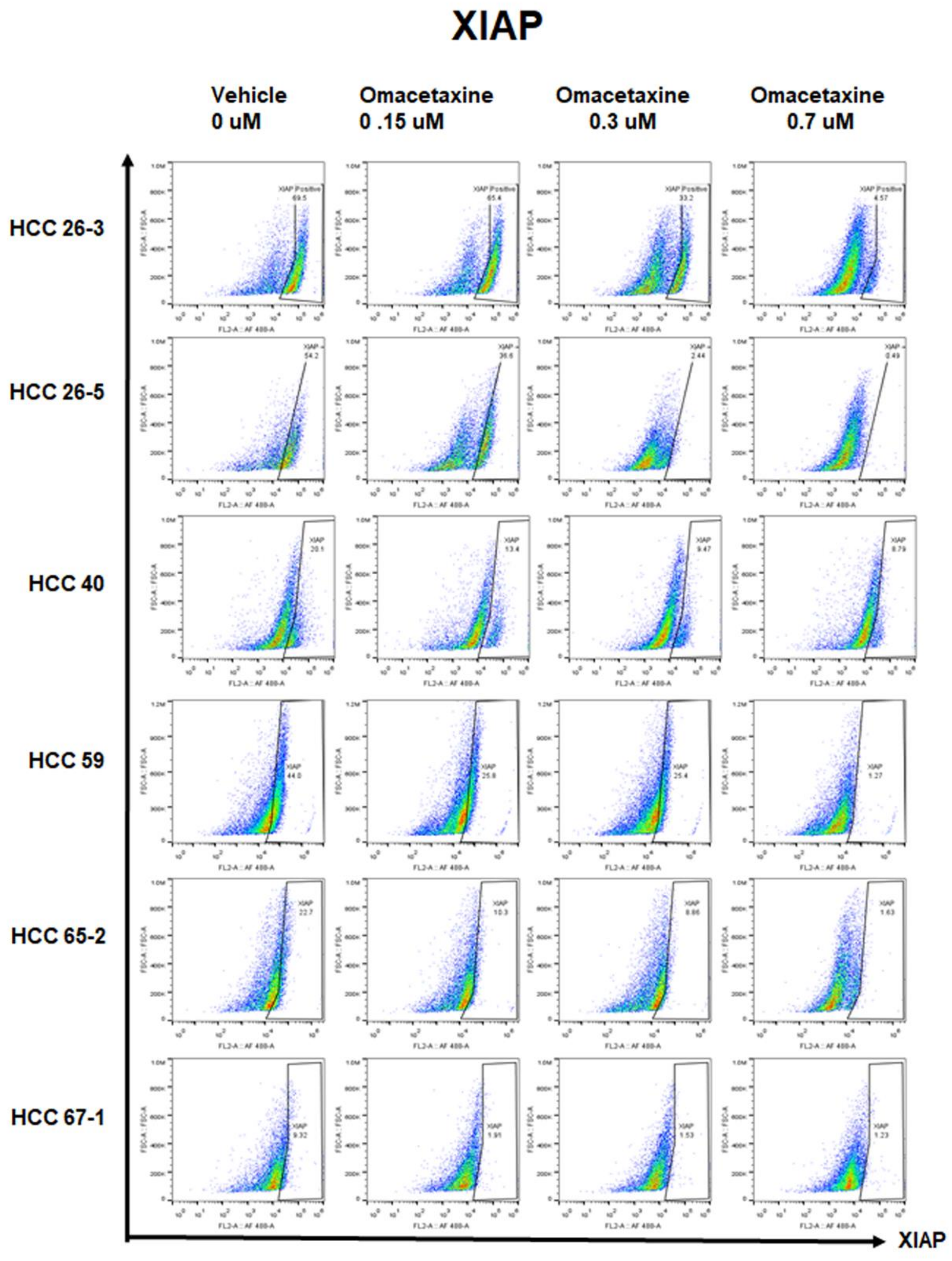

Supplementary Fig. 10. Flow cytometry gating plots data for XIAP in 6 HCC PDOs.

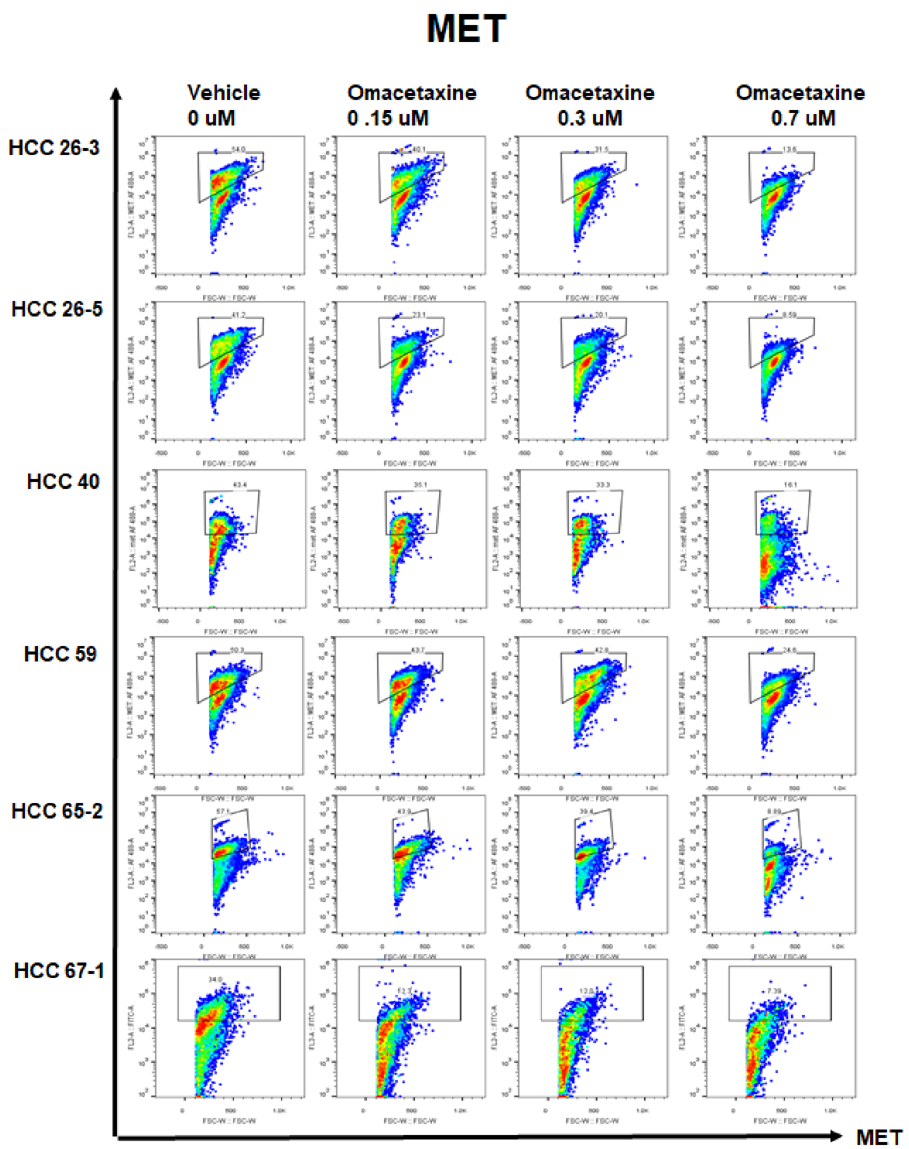

Supplementary Fig. 11. Flow cytometry gating plots data for MET in 6 HCC PDOs.

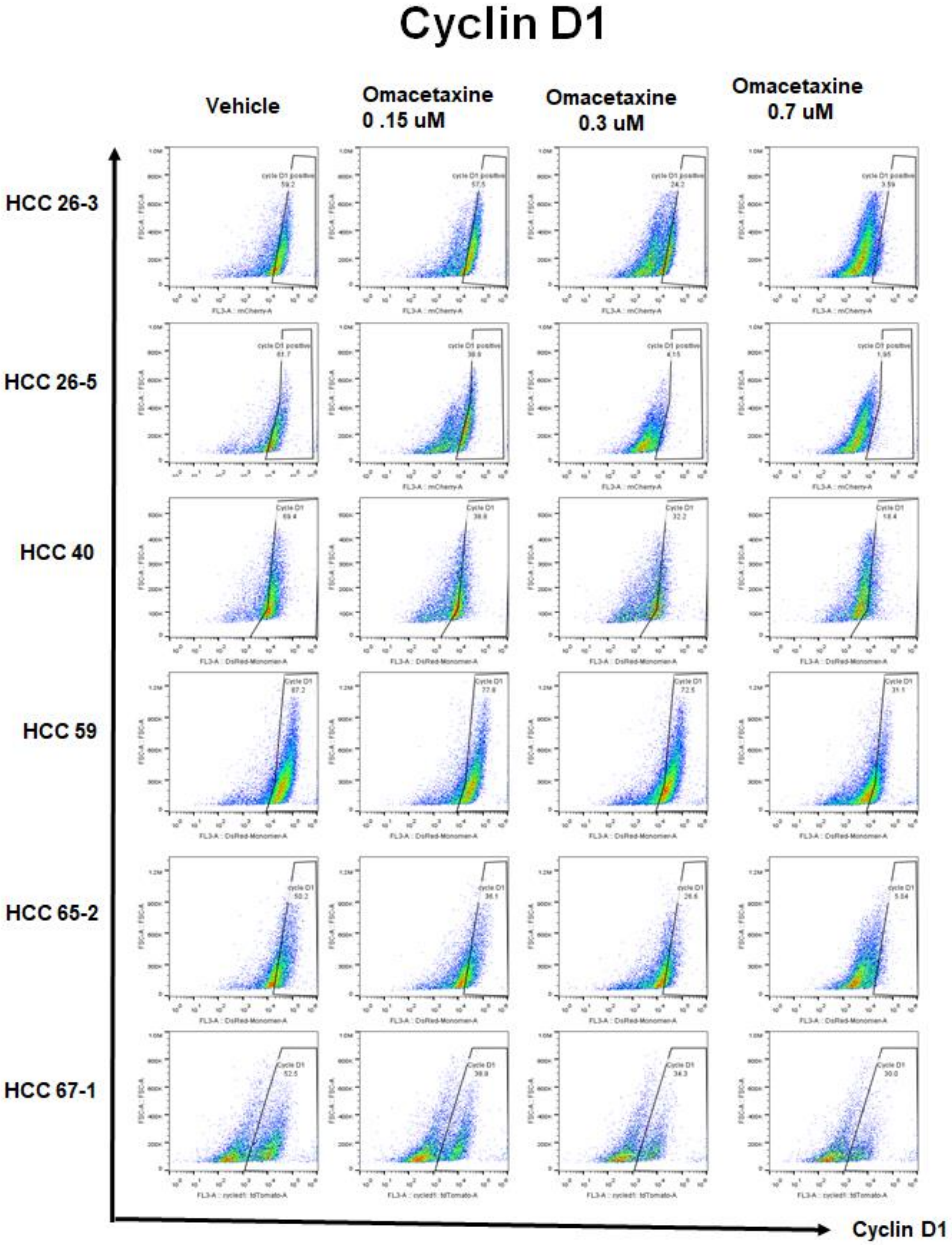

Supplementary Fig. 12. Flow cytometry gating plots data for Cyclin D1 in 6 HCC PDOs.
